# Antiviral treatment perspective against Borna disease virus 1 infection in major depression: a double-blind placebo-controlled randomized clinical trial

**DOI:** 10.1186/s40360-020-0391-x

**Published:** 2020-02-17

**Authors:** Detlef E. Dietrich, Liv Bode, Carsten W. Spannhuth, Hartmut Hecker, Hanns Ludwig, Hinderk M. Emrich

**Affiliations:** 1 Department of Psychiatry, Burghof-Clinic, Ritterstr. 19, 31737 Rinteln, Germany; 2 Center for Systems Neuroscience, Bünteweg 2, 30559 Hanover, Germany; 3 Department of Mental Health, Hannover Medical School, Carl-Neuberg-Str. 1, 30625 Hanover, Germany; 4 Joint Senior Scientists, Freelance Bornavirus Workgroup, Beerenstr. 41, 14163 Berlin, Germany; 5 Department of Biometrics, Hannover Medical School, Carl-Neuberg-Str. 1, 30625 Hanover, Germany

**Keywords:** Major depression, bipolar disorder, Borna disease virus 1 (BDV-1), antiviral treatment, amantadine, double-blind placebo-controlled randomized clinical trial (RCT)

## Abstract

**Background:**

Whether Borna disease virus (BDV-1) is a human pathogen remained controversial until recent encephalitis cases showed BDV-1 infection could even be deadly. This called to mind previous evidence for an infectious contribution of BDV-1 to mental disorders. Pilot open trials suggested that BDV-1 infected depressed patients benefitted from antiviral therapy with a licensed drug (amantadine) which also tested sensitive in vitro. Here, we designed a double-blind placebo-controlled randomized clinical trial (RCT) which cross-linked depression and BDV-1 infection, addressing both the antidepressant and antiviral efficacy of amantadine.

**Methods:**

The interventional phase II RCT (two 7-weeks-treatment periods and a 12-months follow-up) at the Hannover Medical School (MHH), Germany, assigned currently depressed BDV-1 infected patients with either major depression (MD; *N* = 23) or bipolar disorder (BD; *N* = 13) to amantadine sulphate (PK-Merz®; twice 100 mg orally daily) or placebo treatment, and contrariwise, respectively. Clinical changes were assessed every 2–3 weeks by the 21-item Hamilton rating scale for depression (HAMD) (total, single, and combined scores). BDV-1 activity was determined accordingly in blood plasma by enzyme immune assays for antigens (PAG), antibodies (AB) and circulating immune complexes (CIC).

**Results:**

Primary outcomes (≥25% HAMD reduction, week 7) were 81.3% amantadine vs. 35.3% placebo responder (*p* = 0.003), a large clinical effect size (ES; Cohen’s d) of 1.046, and excellent drug tolerance. Amantadine was safe reducing suicidal behaviour in the first 2 weeks. Pre-treatment maximum infection levels were predictive of clinical improvement (AB, *p* = 0.001; PAG, *p* = 0.026; HAMD week 7). Respective PAG and CIC levels correlated with AB reduction (*p* = 0,001 and *p* = 0.034, respectively). Follow-up benefits (12 months) correlated with dropped cumulative infection measures over time (*p* < 0.001). In vitro, amantadine concentrations as low as 2.4–10 ng/mL (50% infection-inhibitory dose) prevented infection with human BDV Hu-H1, while closely related memantine failed up to 100,000-fold higher concentration (200 μg/mL).

**Conclusions:**

Our findings indicate profound antidepressant efficacy of safe oral amantadine treatment, paralleling antiviral effects at various infection levels. This not only supports the paradigm of a link of BDV-1 infection and depression. It provides a novel possibly practice-changing low cost mental health care perspective for depressed BDV-1-infected patients addressing global needs.

**Trial registration:**

The trial was retrospectively registered in the German Clinical Trials Registry on 04th of March 2015. The trial ID is DRKS00007649; https://www.drks.de/drks_web/setLocale_EN.do

## Background

Mental health was given a global health priority by the World Health Assembly in 2013 [1]. As many as 700 million people (7.4%) were affected by mental disorders according to the 2010 Global Burden of Disease Study, accounting for both a huge individual and societal burden, with societal costs of $2.5 billion in 2010, and projections of $6 billion by 2030 [1]. Depressive disorders contributed to the largest proportion (40.5%) of disability-adjusted life years (DALYs) within mental diseases. They accounted for almost one third of the global suicide burden (10.4 of 36.2 million DALYs), over 80% attributable to low and middle income countries [2].

Antidepressants are first-line treatments for depression at least in high-income countries. Up to recent generation antidepressants, serotonin reuptake inhibitors (SSRIs), they are all based on the concept of monoamine deficiency in depression, and therefore increase levels of serotonin, norepinephrine and/or dopamine through different mechanisms. However, their limited efficacy has been under intense debate [3], and patients continue to experience low remission rates, delayed therapeutic onset, limited effectiveness in milder depression, intolerability and relapses [4]. The potential antidepressant effects of amantadine, an old enigmatic drug, have been addressed suggesting a versatile pharmacodynamics profile, but whether it acts through low-affinity non-competitive N-methyl-D-aspartate (NMDA) receptor antagonisms targeting glutamate, or other/combined modes, remained to be determined [5]. A completely novel capacity of amantadine as antiviral compound against human Borna disease virus (BDV-1) strains coincided with antidepressant effects in a BDV-infected patient with therapy-resistant bipolar depression [6]. This serendipitous discovery and further supportive open trials [7, 8] led us to conduct a “proof of concept” randomized double-blind placebo-controlled cross-over trial reported here. The novel rationale included a paradigm shift which cross-linked both depression with BDV-1 infection, and anti-BDV capacity of amantadine with antidepressant efficacy in infected patients.

Bornaviruses are unique in that they are evolutionary ancient non-segmented negative-strand ribonucleic acid (RNA) viruses (order *Mononegavirales*; family *Bornaviridae*) with nuclear replication [9], covering a host spectrum from reptiles to mammals (> 25% genetic divergence). Only BDV-1, the evolutionarily youngest prototype of the species *Mammalian 1 bornavirus* [10], has made it around the globe. BDV-1 strains (classical BDV-1 in humans and mammalian animals) have highly conserved RNA genomes (< 5% divergence) [11, 12], differing largely from a variegated squirrel 1 bornavirus (VSBV-1) which was proposed to underlie three human cases of fatal viral encephalitis in highly exposed squirrel breeders [13]. Classical BDV-1 strains are non-cytolytic, have target cells in brain and blood establishing life-long persistence, and share the ability to cause neurologic and behavioural disorders in mammalian hosts [14]. Although the majority of infections follows a sub-clinical course [15], even deadly outcomes are possible triggered by impaired immune defence [16, 17]. Unexpectedly, BDV-1 caused fatal encephalitis recently occurred in transplant recipients who had received organs from a BDV-1 infected healthy donor [18], and another case was reported unrelated to transplantation [19].

The “mood virus hypothesis” of depression is supported but as yet not confirmed by linking unique BDV properties with lines of evidence from human infection [15–17], namely virus isolates and infection prevalence. Human viruses recovered from psychiatric patients’ peripheral blood mononuclear cells cells (PBMCs) [20] and brain [21], were proven to be authentic through marked biological differences to animal viruses [22, 23], despite close genetic relationship [24]. Their acknowledgement was, however, constrained by misconception [25]. Serum antibodies (AB) and BDV-specific RNA in PBMCs worldwide indicated higher infection prevalence of psychiatric patients than controls in many but not all studies [26–38]. Failure of detection of any these markers in psychiatric patients occurred as well [39]. A recent meta-analysis indicated a 3.25 times higher likelihood of BDV infection for depressed than healthy people [40]. However, comparability was poor due to differing sensitivity levels of antibody and RNA techniques.

The discovery of circulating immune complexes (CIC) in blood plasma [41] explained that in any BDV-1 infected host, most of plasma AB and antigens (N and P protein; N/P dimers) (PAG) are bound within CIC, whereas unbound AB as well as PAG are less frequent at the same time.

Our novel RCT rationale aimed to evaluate both the antidepressant and antiviral efficacy of amantadine vs. placebo. Longitudinal clinical profiling mainly by the 21-item Hamilton rating scale for depression (HAMD) [42] was paralleled by BDV-1 infection profiling, allowing for the simultaneous quantitative determination of CIC, PAG, and AB through a modular enzyme-immune-assay (EIA) technique [41]. The rationale of a mainly antiviral mode of action for amantadine (1-aminoadamantane) was addressed through in vitro efficacy studies in comparison with the closely related derivative memantine (1-amino-3,5-dimethyladamantane).

## Methods

### Study Design

**Fig. 1:**
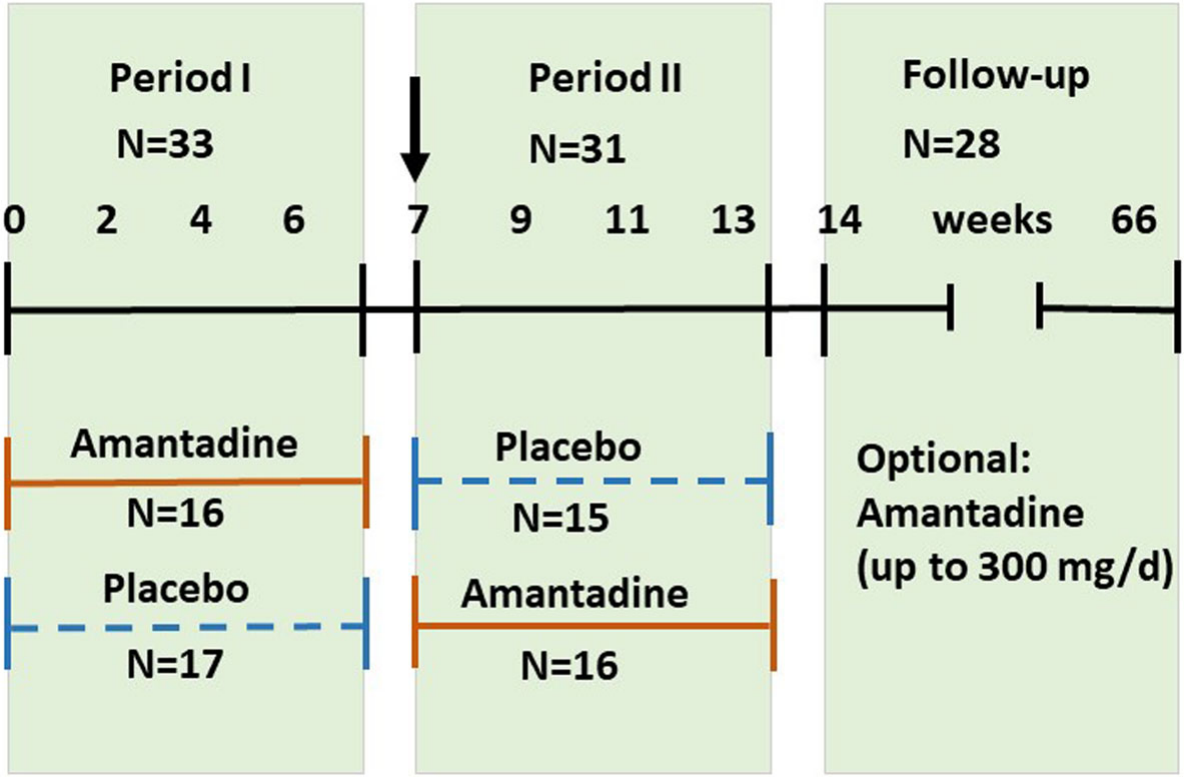
Study design. The graphic illustrates the timeline of the two treatment periods and the follow-up, as well as the cross-over design of intervention by either amantadine or placebo and vice versa of the study, and indicates the number of patients who finished each period (N)

The randomized clinical trial (RCT) was designed as an interventional phase II mono-centre double-blind placebo-controlled cross-over study followed by a 12-months follow-up period (Fig. 1). The cross-over design was an ethical request due to previously beneficial open trials [6–8] and guaranteed that all patients received the same overall treatment by end of the trial. All patients gave written informed consent prior to their participation in the study. The RCT was registered retrospectively on 04th of March 2015 in the German Clinical Trials Registry under the registration ID “DRKS00007649” (see Additional file 1: Trial registration), and was approved by the local Ethics Committee (Reference No. 1508–1997) of the Hannover Medical School (MHH), Hanover, Germany (see Additional file 2: Study history and disclaimer).

### Patients

**Fig. 2:**
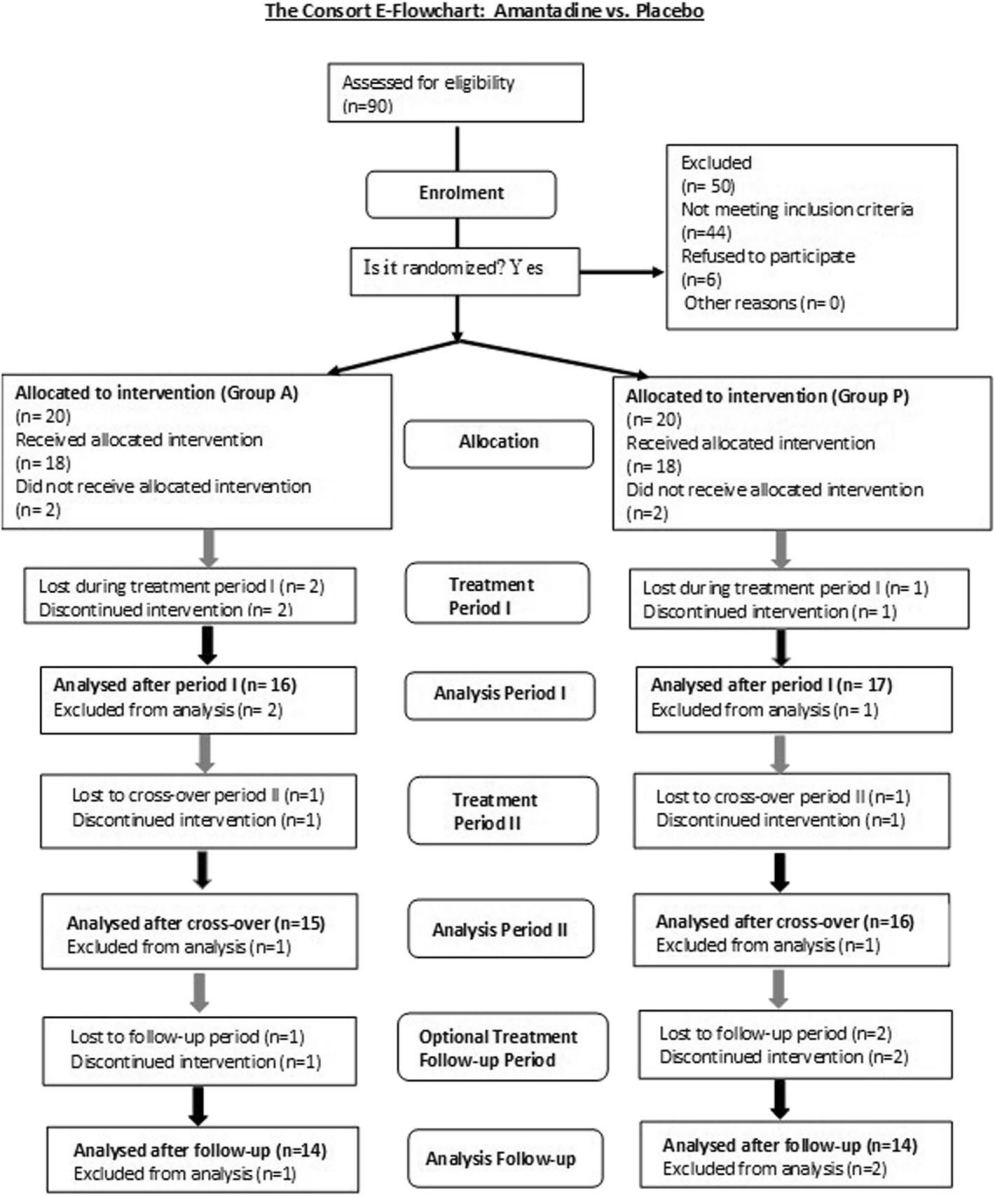
CONSORT Flow Diagram. The CONSORT flow chart illustrates the enrolment of the patients, their allocation to intervention groups in treatment period I, period II, and optional treatment in the follow-up period, as well as the finally analysed patients

All patients were kept informed of all study details including the cross-over design. Written informed consent was given by all patients of the RCT. Recruitment and allocation of patients throughout the clinical trial are summarized in Fig. 2. Of 90 patients assessed for eligibility at the Department of Clinical Psychiatry and Psychotherapy, MHH, Hanover, Germany, 40 were enrolled. Inclusion criteria were BDV-1 infection and a current depressive episode. Infection variables were determined at the Robert Koch Institute, Berlin, Germany for at least two consecutive time-points prior to study entry (see Additional file 2: Study history and disclaimer). Of 36 patients allocated to intervention, 18 each were randomly assigned to either Group A (amantadine) or Group P (placebo) in period I (weeks 1–7; 6 weeks treatment, 1 week wash-out) of the trial. Amantadine sulphate (totally 200 mg) was given orally and twice daily (100 mg/morning and 100 mg/midday). Two patients of Group A and one patient of Group P discontinued intervention in period I. For the cross-over period II (weeks 8–14), 16 remaining patients of Group A received placebo and 17 remaining patients of Group P received amantadine (totally 33 patients). One patient of either group discontinued intervention in period II, leaving 15 patients of Group A and 16 patients of Group P, who completed the cross-over treatment (totally *N* = 31). The post-trial follow-up period of 12 months with optional amantadine treatment up to 300 mg/d was completed by 28 remaining patients. A dosage adaptation was offered to adjust to 2–4 mg amantadine daily per kilogram (kg) body weight (BW) reflecting mean daily dose ranges of amantadine of 100–200 mg for patients between 50 kg and 75 kg BW and 150–300 mg for patients with more than 75 kg BW.

Of the 33 patients finishing the first treatment period (7 weeks), 60% (*N* = 20) were diagnosed as having recurrent (at least one former affective episode documented) major depression (MD), and 40% (*N* = 13) as having bipolar depression (BD; bipolar I, *N* = 7; bipolar II, *N* = 6), according to DSM-IV [43], since a mean of 15.1 ± 10 years. They were of either sex and between 33 and 69 years old. Severity of depression at baseline according to HAMD [42] was 12–20 points for outpatients and 15–30 points for inpatients. Severity differences were unrelated to patients’ age, sex, socioeconomic status, and educational years. Out- and inpatients were randomly assigned to either Group A (amantadine) or Group P (placebo).

Exclusion criteria were major organic diseases, a history of intolerance of compounds of PK-Merz® (amantadine sulphate), minor depression, current substance dependence, presence of any central nervous system, neuromuscular or uncontrolled systemic and other severe medical disorders, psychotic features or serious suicidal risks, and pregnancy. Exclusion of organic diseases were done by routine medical examinations and blood tests. Therapeutic pre-treatment strategies (N = 20) were maintained and remained unchanged during the study. An overview on baseline characteristics of study patients is given in Table 1.

**Table 1:** Baseline characteristics of patients

Characteristic	Amantadine	Placebo	Total	*P*-value
	(*N* = 16)	(*N* = 17)	(*N* = 33)	
Age, mean ± SD, years	50.69 ± 10.18	54.67 ± 9.63	52.79 ± 9.94	0.154
Female, N (%)	12 (75)	8 (47.1)	21	
> 1 year of college, N (%)	14 (87.5)	15 (88.2)	29	
Recurrent major depression	10 (62.5)	10 (58.8)	20	
Bipolar depression, N (%)				
Bipolar I	3 (18.75)	4 (23.5)	7	
Bipolar II	3 (18.75)	3 (17.6)	6	
Mean number of depressive episodes per year (past 3 years prior to current episode)	1.0 ± 1.1	1.04 ± 1.2	1.02 ± 1.1	0.904
Mean duration of depressive episodes (weeks) (past 3 years prior to current episode)	25.6 ± 21	23.7 ± 18	24.6 ± 19	0.781
Duration of illness (years)	12.4 ± 9	17.6 ± 11	15.1 ± 10	0.135
Patients with antidepressant or mood stabilizing co-medication, N (%)	12 (75)	8 (47.1)	20	
Amitriptyline	3	3	6	
Doxepin	1	1	2	
Mirtazapine	1		1	
Clomipramine	1		1	
Trimipramine	1		1	
Tranylcypromine	1		1	
Moclobemide	1		1	
Sertraline		2	2	
Paroxetine	1	1	2	
Dibenzepine	1		1	
Maprotiline		1	1	
Lithium	2	2	4	
Carbamazepine	2	2	4	
Valproate	1		1	
Lorazepam	3	1	4	
Alprazolam	1		1	
Zolpidem		1	1	
Sulpirid	1		1	
L-Thyroxin		2	2	
Dihydroergotamine	1		1	
Estrogenes	1	1	2	

### Clinical assessments

#### Definition of outcomes

The Primary Outcome was defined as change of depressive symptoms measured by total HAMD (21 items) [42] after 6 weeks of treatment plus 1 week wash out, comparing amantadine and placebo groups. Clinical endpoints were defined as reduction of pre-treatment HAMD scores of ≥25% by week 7 (end of period I). Additionally and in parallel, the change of BDV-1 activity was studied (see Infection assessments). According to the trial protocol, the cross-over therapy switch after 7 weeks was designed for ethical reasons to ensure that all patients benefit from the putative efficacy of amantadine. A statistical in-depth analysis of cross-over results was not taken under consideration, because carry-over effects of the first treatment period could not be excluded despite the 1 week wash out.

The Secondary Outcomes were defined as changes in additional standardized depression inventories after 6 weeks of treatment plus 1 week wash out, comparing amantadine and placebo groups. The depression related well-being status was measured by the mental health self-rating score „Befindlichkeitsskala nach von Zerssen “(BfS) [44]. HAMD and BfS rating scales were evaluated at baseline and weeks 2, 4, 7, 9, 11, 13, 14. Further symptoms, e.g. somatization, were rated on the self-rating symptoms check-list (SCL-90R) [45] at baseline and weeks 2, 7, 9, 14.

#### Evaluation measures (clinical outcomes)

Assessment and documentation of depression- related and other symptoms were made blind with regard to treatment arms (amantadine or placebo). After finishing periods I and II the patients were offered to be followed-up in an open setting with optional amantadine treatment for at least 12 months in between which all clinical and infection variables were analysed again. The clinical course (HAMD, 21-items) in each treatment period was analysed through one measure for baseline and 12-month follow-up investigation, and the maximum (MAX) and mean (MEAN) of two different measures during the first (week 4 and 7), and the second treatment period (week 11 and 14).

### Infection assessments

#### Detection methods

BDV-1 infection was monitored by quantitative determination of specific circulating immune complexes (CIC), antigens (PAG), and antibodies (AB) in blood plasma using a modular enzyme-immune-assay (EIA) technique [41]. Test specificity based on two monoclonal antibodies (mABs) (anti-N W1; anti-P Kfu2) [46] had been validated through epitope mapping, and test sensitivity by recombinant proteins [47]. The EIA-systems were applied throughout the study as for the superiority of antigen/antibody-related assays over nucleic acid-related techniques. All laboratory investigations were made blind for any information about treatment arms and/or clinical outcomes in that samples were coded in 5-digit numbers upon receipt. Initial sample processing of citrated blood samples included separation into plasma and PBMCs by density gradient centrifugation (Ficoll-Paque), coding prior to any assay, and storage at − 20 °C and − 80 °C, respectively. Initial handling and assay performance was done by different laboratory personnel. Raw assay data collection and formal verification (independent control samples) were solely made on coded samples, blinded to any clinical information.

Antigens (N- and P-protein; N/P heterodimers) were basically determined from plasma (PAG) as predominant source, using an initial dilution of 1:2. Antibody bound antigen (CIC) was determined from plasma using an initial dilution of 1:20, and antibodies (AB) using an initial dilution of 1:100 in respective EIAs. Antibodies were additionally determined by an indirect immunofluorescence test (IFT) at initial dilutions of 1:10, using a double-stain technique as described previously [48]. All EIAs used the same standardized cut off value of ≤0.1. The cut off has been calculated as mean value of negative samples plus three standard deviations.

#### Conducted tests

A total of N = 2947 single-item tests were conducted, covering the above assays and 35 study patients (including two drop-outs). A mean number of 16.8 samples per patient were examined over time, adding up to a mean of totally 588 tests in each assay.

#### Evaluation measures (infection)

Infection variables were assessed without knowledge of the clinical outcome. CIC, antigens and antibodies were analysed by maximum (MAX) and mean (MEAN) measures of each variable as well as of summated measures. Assessments included at least two pre-treatment scores at baseline, at week 4 and 7 for the first, and week 11 and 14 for the second treatment period, respectively. For the 12-month follow-up investigation only one set of single and summated BDV measures was available.

### In vitro assays

We applied two different methods to quantitatively determine the antiviral efficacy of amantadine in vitro which were in part described previously [6, 15].

#### In vitro prevention of infection

These assays used uninfected young rabbit spleen (YRS) cells grown in 24-well tissue culture plates according to standard procedures [46]. Amantadine sulphate (4 mg/mL) was dissolved in ethanol, diluted 1:10 in phosphate-buffered saline (PBS, pH 7.2), and applied in geometric dilution steps (base 2) to YRS cells, starting with 40 μg/mL for 1 h at 37 °C. Thereafter, cells were infected with 100 focus forming units (FFU) of human BDV strain Hu-H1 (passage 55) [20]. After 2 h at 37 °C cells were washed with medium and kept for 6 days under standard conditions (37 °C, 5% CO_2_, 100% humidity). Foci were determined for each culture by standard cell-ELISA as described previously [46]. Buffer control indicated 100% infection (0% prevention of infection). Exactly the same protocol and virus strain were used to conduct the parallel assay with memantine, here starting with an initial drug concentration of 200 μg/mL. The efficacy of amantadine and memantine to prevent BDV-1 infection was compared according to the 50% infection-inhibitory dose (ID _50_).

#### In vitro inhibition of replication

These longitudinal assays used human oligodendroglial (OL) cells persistently infected with either the above human strain BDV Hu-H1 [20] or the non-natural laboratory adapted animal strain V [14]. For a treatment period of 60 days, cells were split every 3 days and each culture was kept at different amantadine doses between 1.2 μg/mL down to zero. At each passage comprising 15 time points within the 2-months period, virus titres were determined by titrating suspensions of 5 × 10^7^ ultra-sonicated OL cells on YRS cells as described earlier [20]. Virus infectious units were expressed as focus forming units (FFU) per mL as already mentioned, providing that one infectious unit causes one focus (20–50 antigen-carrying cells) which can be visualized by a focus immunoassay (cell ELISA) [46]. The efficacy of amantadine to inhibit replication was analysed in accord to dose and time, comparing two strains of different origin and biology [22, 23].

### Statistical analysis

#### Clinical outcomes

According to the primary and secondary outcomes (change of depressive symptoms) t-tests were applied to compare HAMD-scores [42] of both treatment groups, using the one-sided version at significance level *p* ≤ 0.05. Analyses included the differences between pre-treatment scores and scores at week 2, 4, 7, and 9 of the HAMD-score (DHAMD), the single HAMD items (1–21), certain clusters of these items indicating melancholic features (“DENDOG”, represented by the HAMD-items 5, 6, 7, 8, 12, 16, and 18) or retardation (“DRETARD”, represented by the HAMD-items 1, 7, 8, and 18) of the patients, and changes of the mental state self-rating score (BfS) [44]. Analyses were based on above evaluation measures representing the clinical course in each treatment period.

A two-sided paired t-test was applied to compare the frequency and duration of depressive episodes (no manic episode occurred) during the follow-up period with a period of 3 years prior to the current depressive episode.

In addition to statistical differences between treatment groups, the magnitude of these differences, namely the effect size (ES) values (Cohen’s d; Hedges’s g) [49, 50] were determined [51] for above multi-item and single item scores to evaluate the clinical significance, allowing for comparison with other RCTs.

#### Infection outcomes

T-tests using the one-sided version at significance level *p* ≤ 0.05 were also applied to analyse inter-assay correlations of infection variables by maximum (MAX) and mean (MEAN) measures of each variable as well as of summated measures at above defined time-points.

#### Correlation of clinical and infection outcomes

To determine correlations between initial BDV measures and clinical changes (relative difference of the Hamilton pre-treatment- and 7-week-scores = RDHAMD7) during the first treatment period, a covariance analysis (ANCOVA) with treatment as factor and initial BDV measures as co-variables was performed. The initial BDV measures used were the maximum (MAX) and mean (MEAN) of at least two BDV measures (for BDV variables antigens, antibodies, and CICs) prior to start of the trial.

Patient-related correlations between the clinical course and virus infection variables were investigated across the different treatment periods using a mixed linear model. The mixed model set the clinical course as dependent variable, the patient as random factor, and treatment and infection data as time-dependent factors or covariates, respectively. Statistical analyses on the strength of association within and between clinical and infection variables were done through calculating Pearson’s correlation coefficient (r) and Spearman’s coefficient of rank correlation (2-tailed) by aid of up-to-date SPSS software, finally completed using version 19.0 (IBM SPSS Statistics for Windows, released 2010).

## Results

This is the first report on the clinical and antiviral efficacy of amantadine in BDV-1 infected depressive patients evaluated in a company-independent RCT and conducted in a placebo-controlled cross-over and double-blind design.

### Clinical outcomes

#### Primary response

The main primary outcome measure and principal clinical objective was defined as the change of the 21-item HAMD score between end and beginning of the first 7-weeks period. The differences between the two treatment groups (A = amantadine, P = placebo) were summarized in Table 2.

**Table 2:** Primary clinical outcomes

Patients *N* = 33	Differences A vs. P^a^	Patients with Amantadine(A) *N* = 16	Patients with Placebo (P) *N* = 17
Response		13	6
Non-response		3	11
Response rate (%)		13/16 = 0.8125 (81.25%)	6/17 = 0.3529 (35.29%)
Statistical significance (defined as *p* < 0.05)	*p* = 0.003		
Absolute response difference	81.25–35.29 = 45.96%		
Relative response (RR)	81.25/35.29 = 2.302		
Odds		13/3 = 4.33	6/11 = 0.55
Odds ratio (OR)	4.33/0.55 = 7.872		
Effect size of difference (expressed as Cohen’s *d*)	*d* = 1.0461		
Effect size of difference (expressed as Hedges’s *g*)	*g* = 1.0175		
Effect size of association (correlation coefficient *r*)	*r* = 0.463		

^a^Primary outcome defined as changes between pre-treatment scores and scores at week 7 of the HAMD (DHAMD). Clinical endpoint defined as reduction of HAMD ≥25%. Time endpoint: 7 weeks

Based on 33 patients (*N* = 16 on amantadine) and clinical endpoints of a HAMD-score reduction of ≥25% as shown in Table 2, significantly different response rates of 81.3% for the amantadine (A) and 35.3% for the placebo (P) group (*p* = 0.003) were obtained by week 7. This corresponded to an effect size (ES; Cohen’s d) [49] of 1.046 which valued the clinical significance to be extraordinarily large. By defining endpoints as HAMD-score reduction of ≥50%, the response rates were 43.8% (A) and 0% (P).

By end of both treatment periods and cross-over, after 14 weeks, the clinical response of all remaining 31 patients was 74.2% (23/31) and 54.8% (17/31), as for above endpoints of ≥25% reduction and ≥ 50% reduction of the HAMD-score, respectively. Finally, by end of the follow-up period of 12 months offering optional amantadine treatment, after 66 weeks, the clinical response of the remaining 28 patients reached 82.1% (23/28) and 64.3% (18/28), as for endpoints of ≥25% reduction and ≥ 50% reduction of the HAMD-score, respectively.

The clinical response of all study periods has also been analysed at the level of individual patients (see Additional file 3: Table S1). Remarkably, amantadine treatment in Period I had a strong impact on the placebo effect in Period II in that 69.2% (9/13) responder in Period I maintained response under placebo in Period II, adding up to 80.0% (12/15) in whole Group A at week 14. In contrast, as for placebo treatment in Period I (response 35.3%), a significantly different responder rate of 68.8% (11/16) was reached after cross-over to amantadine in whole Group P in Period II, at week 14. Previous open trials had revealed similar rates after 12 weeks [7, 8]. Of those patients who were offered optional amantadine treatment for 12 further months, almost all (92.9%) (13/14) became responder (Group P).

#### Secondary outcomes and clinical course of treatment

**Fig. 3:**
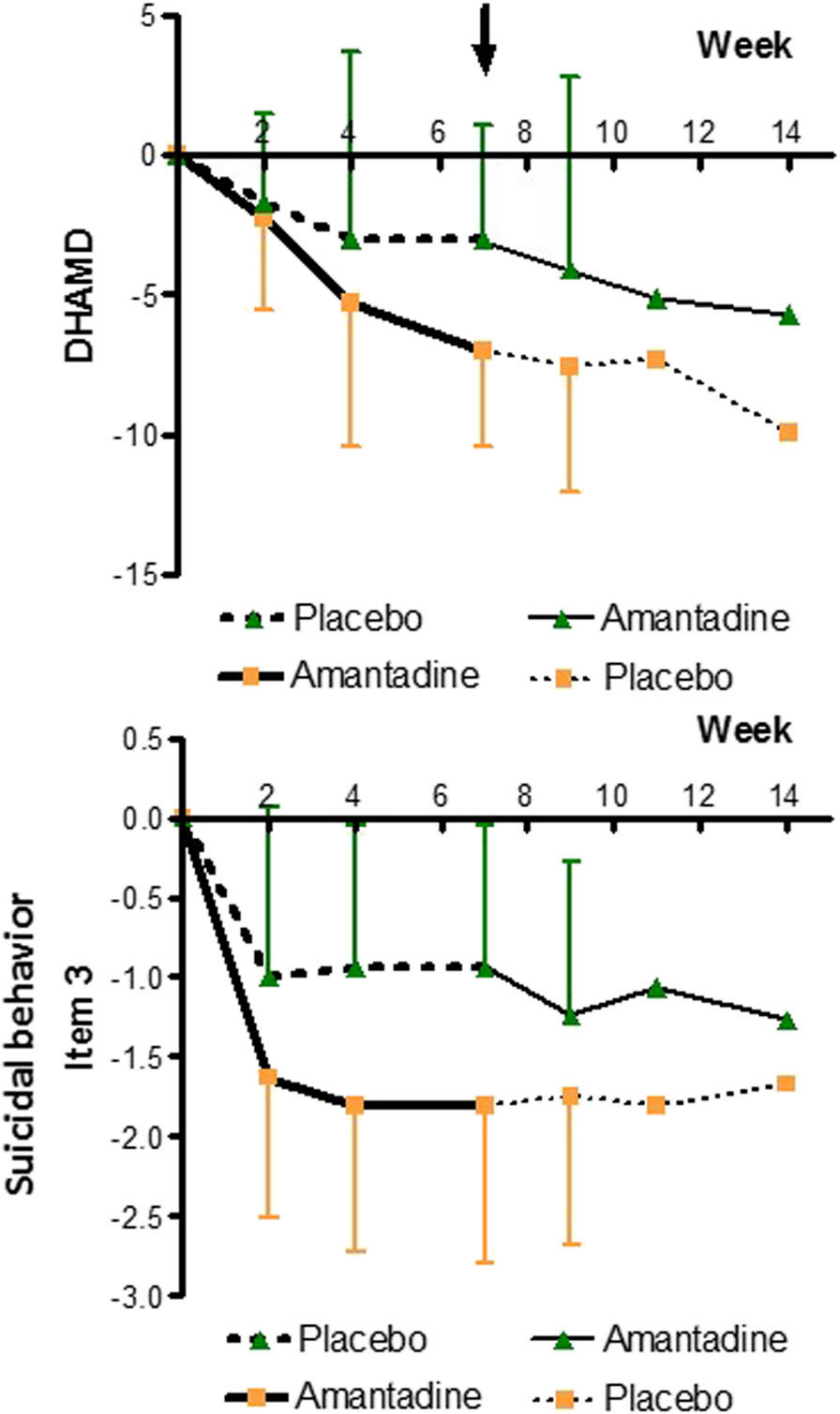
Course of treatment 1 (HAMD-21 and suicidal behaviour, item 3). At the top: treatment effect of amantadine compared to placebo measured by the difference in total HAMD-score, week 7 marked by an arrow. At the bottom: treatment effect by the difference in HAMD item 3 “suicidal behaviour”. For *p*-values and effect size indicators at different time points, see Table 3 and Table 4

**Fig. 4:**
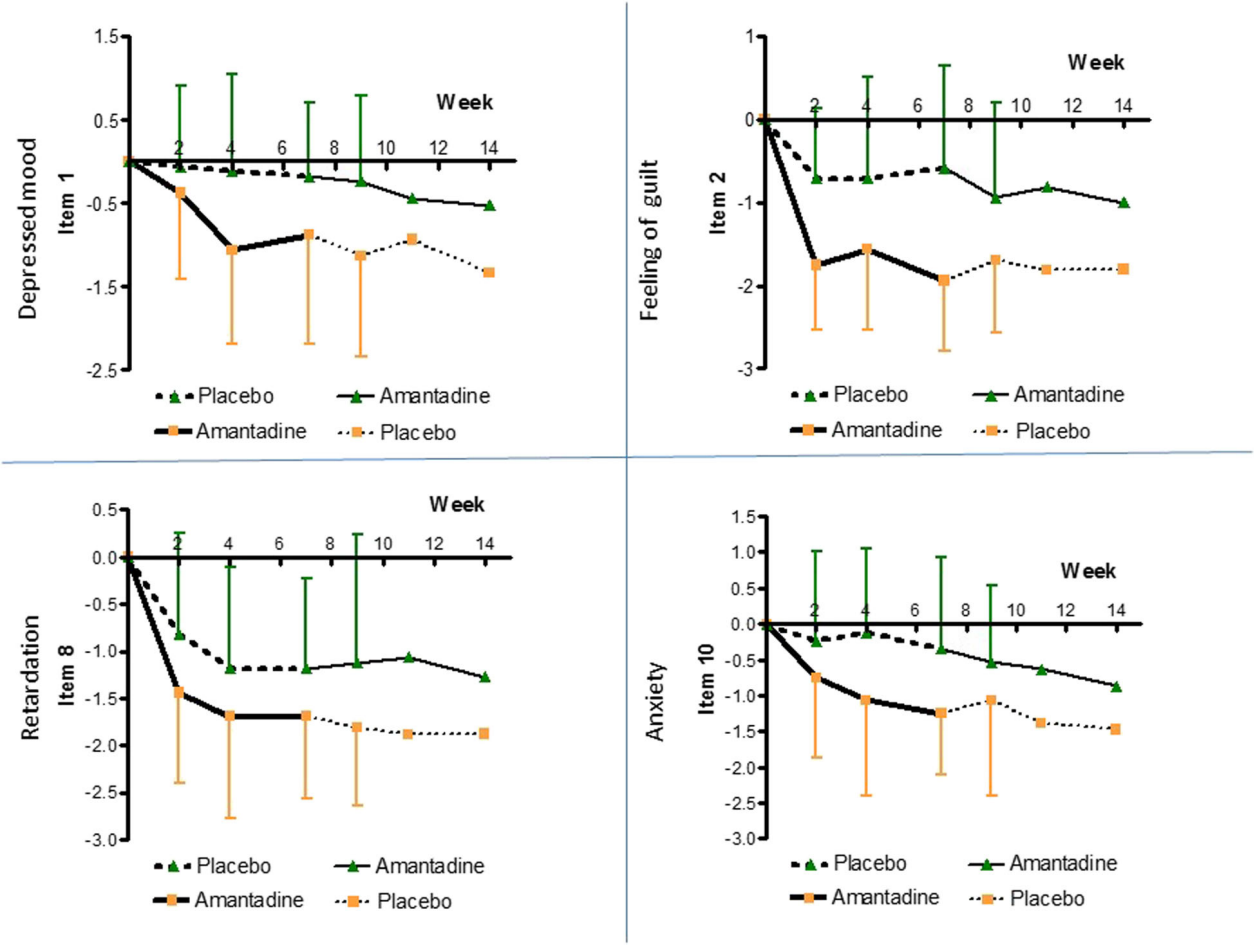
Course of treatment 2 (HAMD items 1, 2, 8, and 10). Treatment effects of amantadine and placebo, respectively, measured by the difference in indicated items 1 (depressed mood), 2 (feeling of guilt), 8 (retardation), and 10 (anxiety) of the HAMD-score. For *p*-values and effect size indicators at different time points see Table 3 and Table 4

**Fig. 5:**
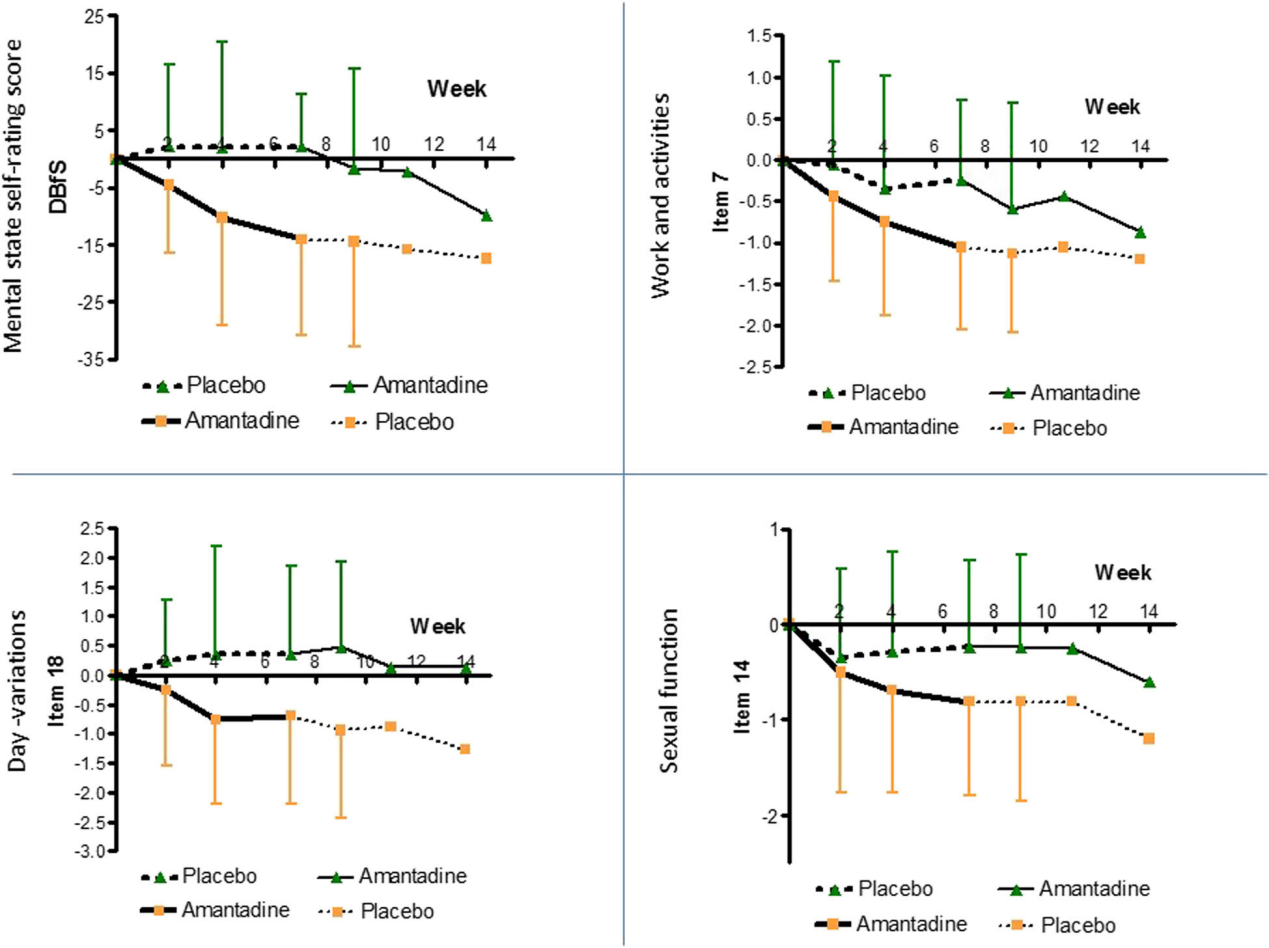
Course of treatment 3 (BfS and HAMD items 7, 18, 14). Treatment effects of amantadine and placebo, respectively, measured by the difference in indicated items 7 (work and activities), 14 (sexual function), and 18 (day-variations) of the HAMD-score, as well as by the self-rated depression-related well-being score (BfS). For *p*-values and effect size indicators at different time points see Table 3 and Table 4

**Fig. 6:**
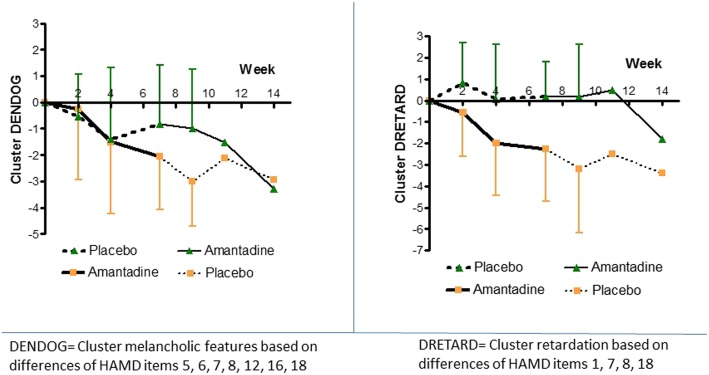
Course of treatment 4 (combined HAMD cluster). Treatment effects of amantadine and placebo, respectively, measured by the difference in combined items of the HAMD-score, clustering for either melancholic (DENDOG) or retardation (DRETARD) features. For *p*-values and effect size indicators at different time points see Table 3 and Table 4

The efficacy of amantadine vs. placebo treatment was monitored throughout the entire study at week 2 to week 14 from baseline. The clinical course of treatment was detailed for total HAMD-21, eight further single HAMD-items and a self-rating score for well-being (BfS), as well as combined HAMD-clusters indicating melancholic and retardation-related features (DENDOG and DRETARD), respectively (Fig. 3, 4, 5 and 6).

With respect to the main clinical outcome after 7 weeks, single-item score differences between amantadine and placebo groups were calculated according to statistical significance, clinical effect size (ES, d-value), and correlation coefficient levels (Tables 3 and 4).

**Table 3:**
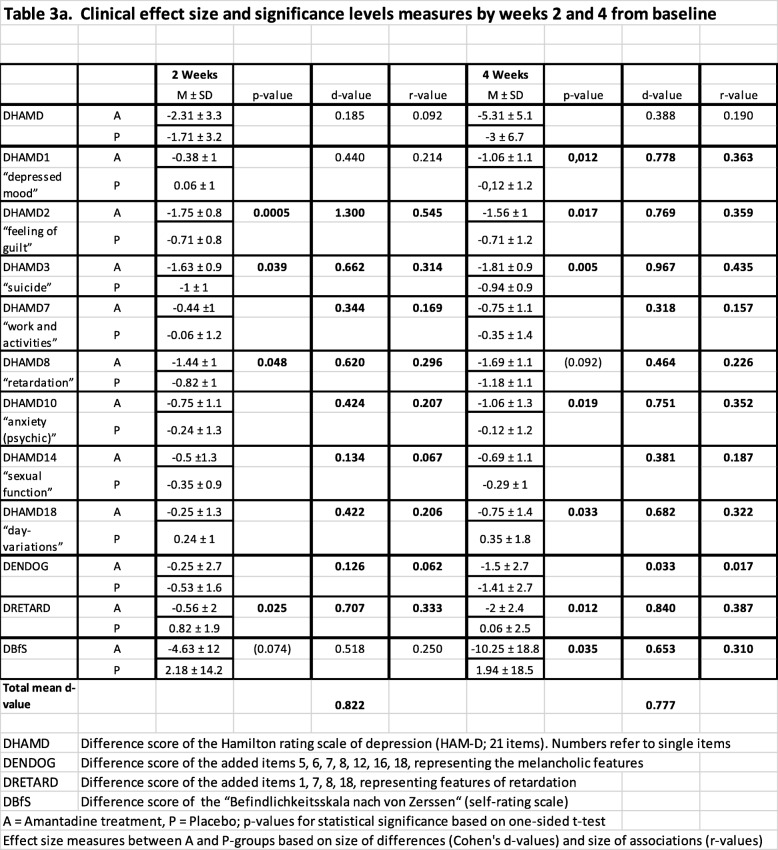
Clinical effect size and significance levels by weeks 2 and 4 from baseline

**Table 4:**
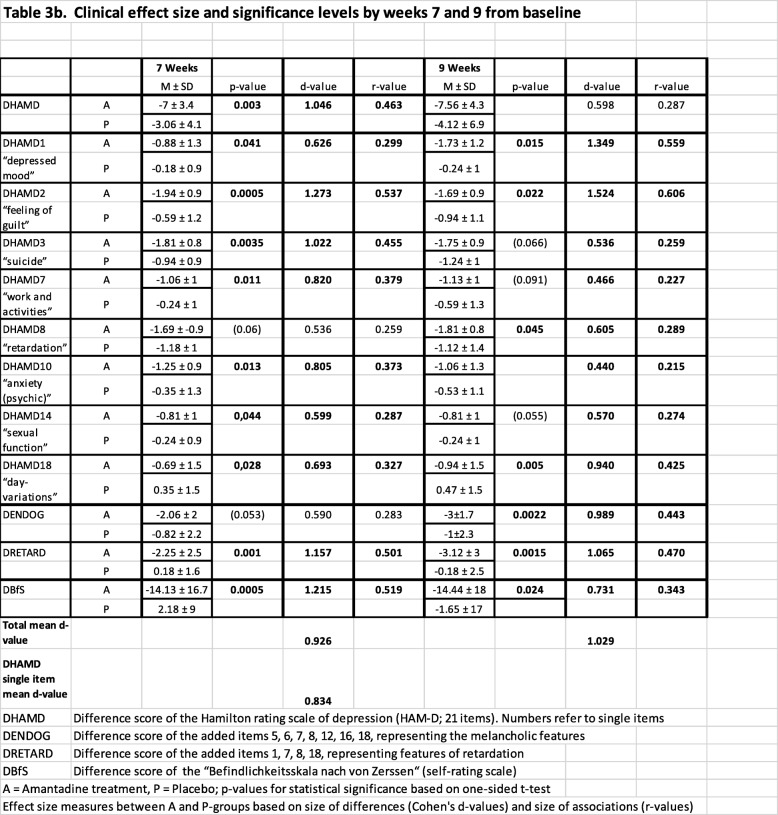
Clinical effect size and significance levels by weeks 7 and 9 from baseline

Notably, as shown in Fig. 3, amantadine significantly reduced “suicidal behaviour” (item 3) as early as by 2 weeks of treatment (*p* = 0.039, d = 0.662), earlier than the total Hamilton score. If this finding holds true in larger RCTs, it could help saving patients’ lives by closing a treatment and safety gap reported for SSRIs [4, 52, 53]. Suicidal behaviour dropped continuously during amantadine treatment, given effect size levels (d-values) of 0.97 by 4 weeks up to 1.02 by 7 weeks.

Likewise by 2 weeks, single HAMD items, namely “feeling of guilt” (item 2; *p* = 0.0005, d = 1.300) and “retardation” (item 8; *p* = 0.048, d = 0.620), were significantly improved by amantadine (Fig. 4, Table 3). By 4 weeks of treatment, also earlier than the total Hamilton score, single items “depressed mood” (item 1; *p* = 0.012, d = 0.778), “anxiety” (item 10; *p* = 0.019, d = 0.751), “day-variations” (item 18; *p* = 0.033, d = 0.682), and the self-rated well-being score BfS (*p* = 0.035, d = 0.653) could be significantly improved by amantadine (Fig. 4 and Fig. 5, Table 3).

The evaluation of combined HAMD clusters representing retardation (DRETARD) and melancholic features (DENDOG) revealed a different time pattern for improvements by amantadine. While the retardation cluster significantly improved as early as by 2 weeks (*p* = 0.025, d = 0.707) and further throughout the course of treatment, melancholic features improved delayed by 9 weeks (*p* = 0.0022, d = 0.989) upon amantadine treatment (Fig. 6, Table 3 and Table 4). Cognitive dysfunction across multiple domains is known as a frequent residual manifestation in depression [54]. Here, we found early and sustained improvement of cognitive impairment by amantadine (DRETARD cluster) providing a remarkable difference to orthodox treatment.

At the end of treatment period I (week 7), amantadine was significantly superior to placebo across all measured single items of HAMD. The pronounced clinical significance of the drug was not only indicated through the large d-value of the total DHAMD-21 difference to placebo (d = 1.05), but also for mean d-values covering depressed mood, feeling of guilt, suicide, work and activities, psychic anxiety, sexual function, and day-variations (mean d = 0.83). The subjective benefit in the amantadine group was best reflected by one of the highest d-values (d = 1.22) in the self-rating score for well-being (DBfS). The cumulative effect size development is additionally provided (Additional file 4: Figure S1).

### Safety

Amantadine at a daily oral dose of 200 mg was very well tolerated confirming previous open trials [6–8]. The treatment emergent adverse events (TEAEs) during the course of treatment periods were evaluated in Table 5. Reported single adverse events were further detailed (Additional file 5: Table S2).

**Table 5:** Treatment emergent adverse events (TEAEs)

Patients N = 32	Differences A vs. P	Patients with Amantadine (A) *N* = 33^a^	Patients with Placebo (P) *N* = 33^a^
Side effects		15	11
No side effects		18	22
Side effect rate (%)	Non-significant difference	15/33 = 0.454 (45.4%)	11/32 = 0.333 (33.3%)
Absolute side effect difference	45.4–33.3% = 12.1%		
Relative risk (RR)	45.4/33.3 = 1.363		
Odds		15/18 = 0.83	11/22 = 0.50
Odds ratio (OR)	0.83/0.5 = 1.66		

^a^Two of the 33 patients did not participate/finish the second treatment period. Time endpoint: 14 weeks, 6 weeks amantadine, 1 week off, 6 weeks placebo, and cross - over treatment

Neither serious adverse events (SAEs) nor significant differences between amantadine and placebo periods were observed. No patient discontinued the treatment due to an adverse event (AE). Notably, none of severe side-effects, such as psychotic symptoms, severe anticholinergic effects or restlessness, and sleeplessness, were reported. Those events, however, had been solely described in the context of intravenous application of amantadine in patients with Parkinson’s disease.

Furthermore, the symptoms-check-list SCL-90R [45], which was used to screen especially for possible somatic symptoms, did not disclose any major differences of the global severity index (GSI) and of eight out of nine subscales. Only the subscale “interpersonal sensitivity” demonstrated a significant difference between the groups (*p* = 0.033), however, related to a reduction of symptoms in the amantadine group (two-sided t-test).

### Sustainability (12-months follow-up)

Besides the above remarkable short-term antidepressant efficacy of amantadine, we could demonstrate sustainability. For the remaining 28 patients who were offered optional post-study amantadine treatment and agreed to a follow-up examination 12 months after end of study, the frequency of episodes was reduced from 1.97 ± 1.6 episodes per year before to 1.0 ± 1.0 episode per year after the two treatment periods (T = 3.525; *p* < 0.002). Likewise, the duration of episodes was reduced from 22.97 ± 19.4 weeks per episode before to 13.07 ± 16.8 weeks per episode after the two treatment periods (T = 4.197; *p* < 0.001). In parallel, the long-term 12-months post-study clinical benefit was significantly correlated with dropped cumulative infection measures (CIC + PAG + AB) over time (*p* < 0.001) (see below).

### Infection outcomes

Active BDV-1 infection relates to proteins (antigen in plasma; PAG), host immune response (antibodies; AB) and circulating immune complexes (CIC), all of which are dynamically interrelated [41].

#### Correlation of pre-treatment infection variables

We found strong significant correlations between MAX and MEAN initial amounts of CIC and AB with antigen (PAG) values (Table 6). No correlation was found between CIC and AB values which may account for the fact that CIC levels are primarily antigen- rather than antibody-driven.

**Table 6:** Correlation of pre-treatment BDV infection variables

			AB		CIC	
PAG			MAX	MEAN	MAX	MEAN
	MAX	*r*	.523	.618	.766	.775
		*p*	.002	.000	.000	.000
	MEAN	*r*	.515	.625	.739	.761
		*p*	.002	.000	.000	.000

Correlation between maximum (MAX) and mean (MEAN) values of initial pre-treatment BDV infection measures (PAG antigen; AB antibodies; CIC circulating immune complexes).

#### Correlation of infection and clinical variables

Considering the abundance of clinical and assay results, a mixed model was applied with the clinical course as dependent variable, the patient as random factor, and treatment and infection variables data as time-dependent covariates, respectively. The data analysis focused on the first treatment period of 7 weeks which was defined as main clinical outcome. An overview on results of in-depth statistical calculations within and between infection variables and clinical outcome are provided in Table 7. Patients’ data of either amantadine or placebo groups were analysed together to provide sufficient statistical power. According to the high clinical effect size of amantadine over placebo, correlations could be mainly assigned to the amantadine group which displayed a clearly more pronounced decrease of infection variables.

**Table 7:**
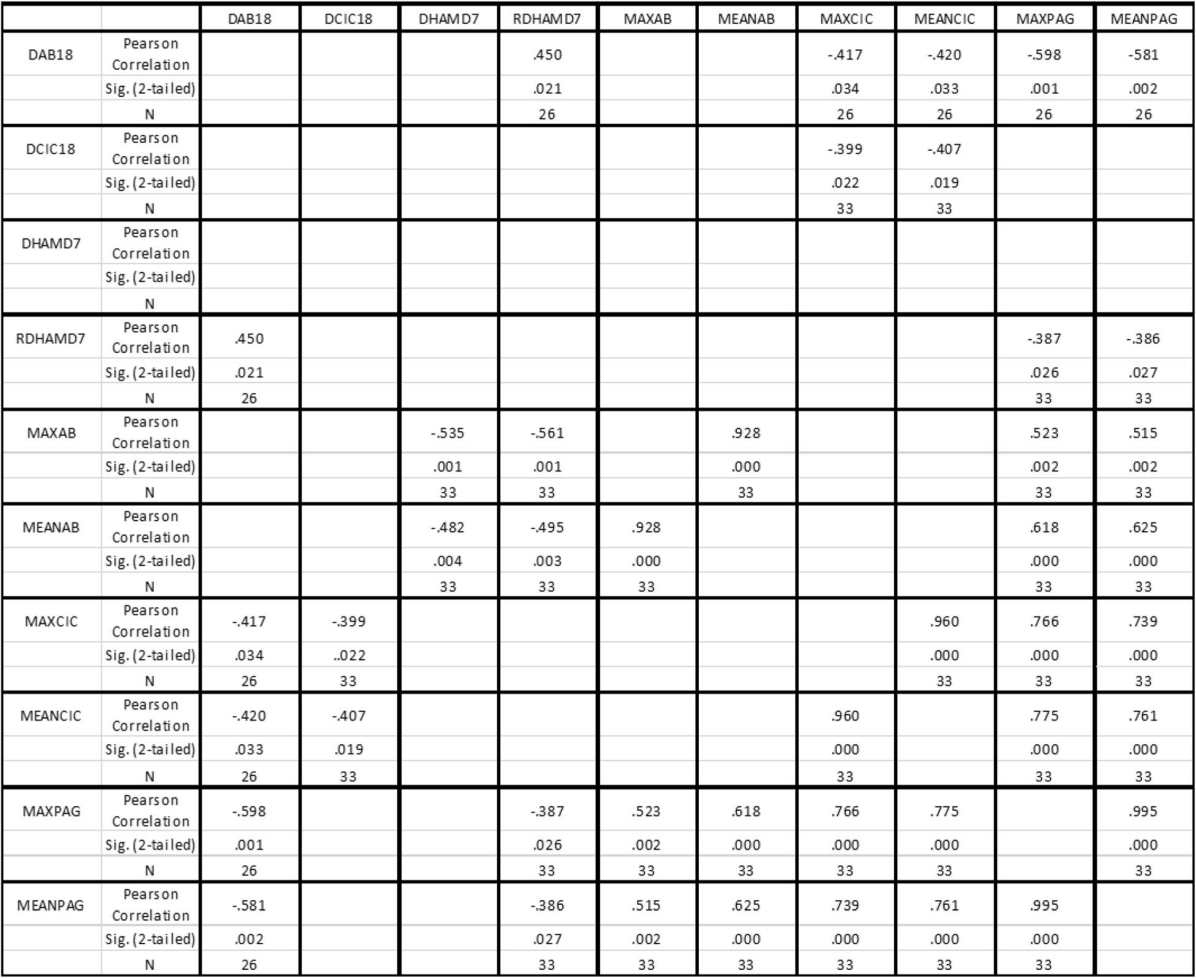
Correlation between infection and clinical variables

Pearson’s correlation coefficient (r) and Spearman’s coefficient of rank correlation (2-tailed) calculated by aid of up-to-date SPSS software were used to statistically determine the strength of association within and between clinical and virus infection variables. For abbreviations and further details, see Methods

Within infection variables**,** MAX and MEAN pre-treatment levels of antigen and CIC correlated significantly with antibody reduction in the first treatment period (MAXPAG *r* = −.598; *p* = 0.001, MEANPAG *r* = −.581; *p* = 0.002; MAXCIC *r* = −.417; *p* = 0.034, MEANCIC *r* = −.420; *p* = 0.033). The higher the initial plasma levels of virus antigen-related variables (PAG and CIC), the more pronounced was the AB reduction in this time period (Table 7; DAB18).

Most remarkable correlations were found between pre-treatment infection variables and favourable clinical outcomes. High initial amounts of antibodies were predictive of pronounced clinical improvement defined as relative HAMD change between baseline (pre-treatment) and week 7 (RDHAMD7) (MAXAB *r* = −.561; *p* = 0.001, and MEANAB *r* = −.495; *p* = 0.003). A strong predictive value of high initial antibodies was also found for pronounced improvement of the self-rating well-being score BfS by week 7. Likewise, initial antigen levels predicted clinical improvement (RDHAMD) both at MAX and MEAN levels (MAXPAG *r* = −.387; *p* = 0.026, and MEANPAG *r* = −.386; *p* = 0.027). In contrast, initial CIC levels displayed no predictive power for the clinical course, but a significant correlation between their MAX and MEAN pre-treatment levels and pronounced CIC reduction by week 7 could be measured (MAXCIC *r* = - .399; *p* = 0.022, and MEANCIC *r* = -.407; *p* = 0.019) (Table 7; DCIC18).

**Fig. 7:**
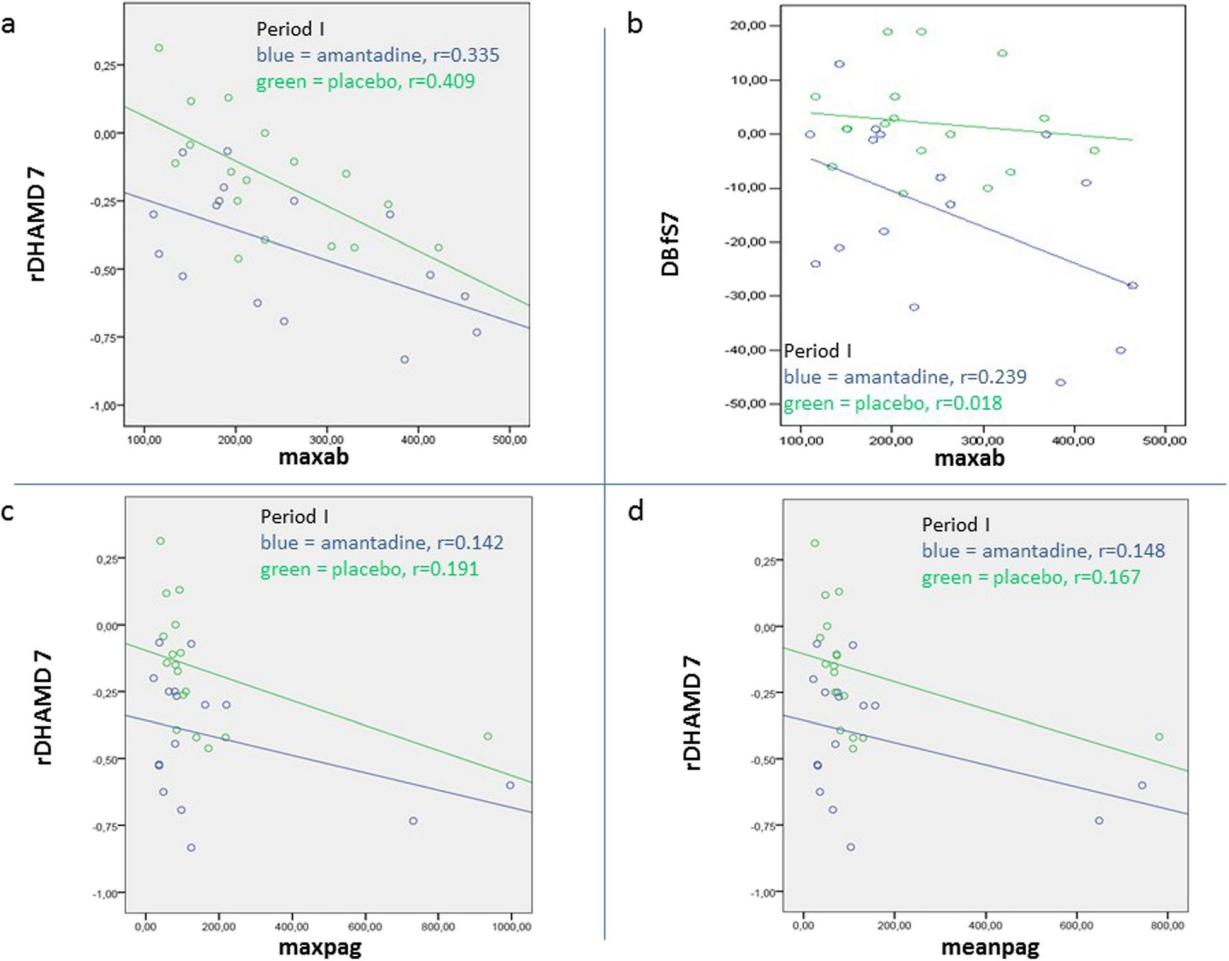
**a**-**d** Pre-treatment infection variables and primary clinical outcome. Pearson’s correlation coefficient of treatment outcome of amantadine and placebo on week 7, as based on differences of the Hamilton score of depression (rDHAMD7) and the self-rated well-being scale (DBfS7) after period I, with pre-treatment values of BDV-1 antibodies (maxab) and antigen (maxpag, meanpag), as indicated in parts (**a**-**d**)

To further highlight treatment outcomes of amantadine and placebo vs. infection in Period I, the relationship of pre-treatment antibody (MAXAB) and antigen levels (MAXPAG, MEANPAG) to HAMD reduction on week 7 (rDHAMD7) as well as of MAXAB and improved self-rated well-being were illustrated (Fig. 7a-d). Correlations of total treatment outcome (rDHAMD7) with maximum pre-treatment loads of either antibody or antigen were additionally provided (Additional file 6: Figure S2 and S3).

#### Sustainability

Clinical improvement in the 12-months post-study follow-up was significantly correlated with dropped overall infection variables over time. A covariance analysis revealed a significant correlation between the mean HAMD-scores and the mean amount of BDV-1 infection scores, when cumulative scores (CIC + PAG + AB) were considered (t = − 3.51; *p* < 0.001).

### In vitro efficacy studies

#### Prevention of infection

**Fig. 8:**
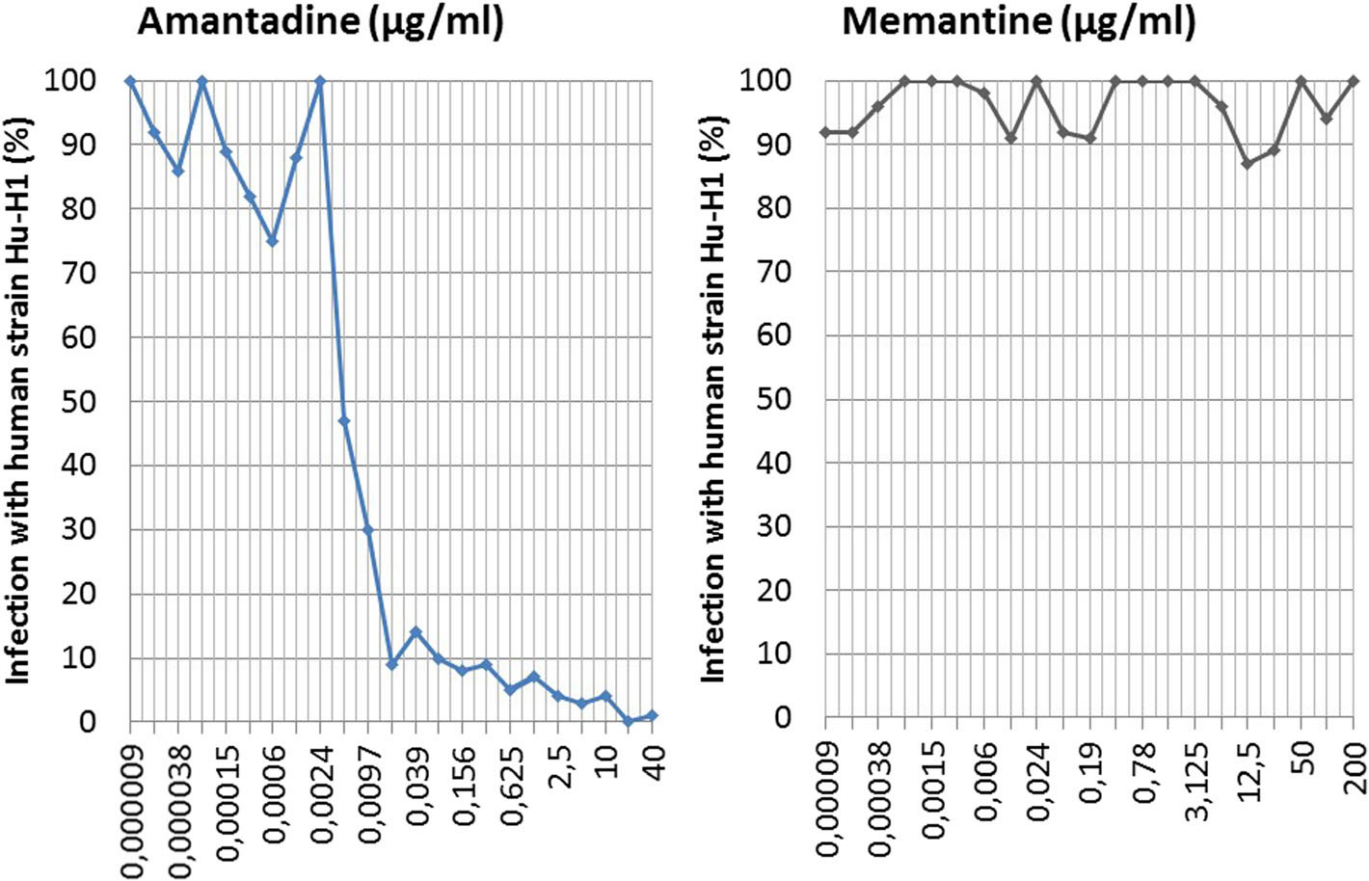
In vitro prevention of infection comparing amantadine and memantine. Prevention-of-infection test by indicated doses of amantadine and memantine in vitro, using young rabbit spleen cells (YRS p108) and human BDV strain Hu-H1 (p55); p = passage

The efficacy of amantadine and memantine to prevent BDV-1 infection was compared according to the 50% infection-inhibitory dose (ID _50_). Based on ID _50_, an amantadine dose as low as 2.4 to 10 ng/mL prevented infection with human strain BDV Hu-H1, while the closely related compound memantine failed up to a 100,000 times higher concentration of 200 μg/mL in the parallel experiment, as demonstrated in Fig. 8.

#### Inhibition of replication

The dose-dependent extraordinary antiviral efficacy of amantadine was further demonstrated in that the in vitro medium dose of amantadine (0.4 μg/mL) which abrogated the above tested human BDV-1 strain by 4 weeks, corresponded to the in vivo blood level resulting from the dose of 200 mg orally per day applied in patients of the here described trial. Notably, the non-natural laboratory strain V was insensitive up to the highest in vitro dose of 1.2 μg/mL (Additional file 7: Figure S4), a finding consistent with the significant biological differences of these two strains [22, 23].

## Discussion

“No health without mental health” stated the World Health Organization (WHO) according to the 2010 Global Burden of Disease Study [1]. The here reported RCT followed a novel rationale which aimed to open a new avenue to safe, cost-effective and sustainable treatment of depressive disorders applicable also in low resource settings. Two provocative hypotheses contested since two decades were combined, namely whether depression could be linked to infection with a putatively mood-modulating virus (BDV-1) [14–17], and whether antiviral capacity of a well-known drug (amantadine) with yet ambiguous modes of action [5] could work as safe and well-tolerated antidepressant in infected patients. Testing an infectious contribution of BDV-1 to mental health, particularly depression, meant to be on a rocky path from the start, because human infection was doubted [25], despite evidence at different levels [20–38]. Recently, human BDV-1 infection became an incontrovertible fact by reports on its ability to even cause fatalities [18, 19], emphasizing the broad capacity of BDV-1 as a human pathogen.

### Clinical outcomes

This first RCT conducted in a placebo-controlled double-blind cross-over design (twice 7 weeks) could validate both antidepressant and antiviral efficacy of amantadine in BDV-1 infected depressed MD or BD patients. The study not only confirmed our 20-years old pilot case report [6], but even outweighed response rates of two similarly sized open trials (68 and 63%, respectively) [7, 8] by achieving a primary response of 81.3% on amantadine vs. 35.3% on placebo after 7 weeks (*p* = 0.003). Our phase II RCT could also confirm that an oral dose of twice 100 mg daily of amantadine sulphate (PK-Merz®) [6–8] is well-tolerated in that neither serious adverse events (SAEs) nor significant SAE differences between amantadine and placebo periods were observed.

What the outcome data revealed were not only a strong statistical difference of DHAMD scores between amantadine and placebo treatment groups, but a pronounced clinical significance according to the large effect size (ES; Cohen’s d) [49] of 1.05 (primary outcome week 7). ES values of 0.2, 0.5, and 0.8 were proposed to represent small, medium, and large effects, respectively [3, 49]. Amantadine efficacy in our trial even tripled overall ES values of 0.32 and 0.31, respectively, found for antidepressants including SSRIs in independent meta-analyses [55, 56]. According to a more recent analysis comparing three widely prescribed SSRIs which revealed a better ES magnitude (0.5) after excluding suboptimal doses [57], our trial came up with at least double ES values. Antidepressants were compromised by selective reporting of positive results [56]. Of seventy-four RCTs on twelve antidepressants involving more than 12,000 patients, studies judged positive by the Food and Drug Administration (FDA) (51%) were 12 times as likely to be published as were studies with non-positive results according to FDA (*p* < 0.001). Furthermore, the overall mean ES values were significantly different between published and unpublished studies reviewed by the FDA (0.37 and 0.15, respectively) [56]. Beyond this criticism, each drug was shown by meta-analysis to be superior to placebo although less than indicated by the literature. Their clinical significance, however, of overall 0.3 is still on debate, based on whether a sharp cut off of 0.5 proposed as medium ES value were used [55] instead of a more continuous measure for drug efficacy [3]. Whatever effect sizes of antidepressants were deemed beneficial in RCTs that of amantadine appeared to outreach them all. Like for total HAMD-21, amantadine was significantly superior to placebo across all measured single items of HAMD, with a large mean ES of 0.834 (week 7) for depressed mood, feeling of guilt, suicidal behaviour, work and activities, psychic anxiety, sexual function, and day-variations.

The risk of suicidal behaviour, especially in the context of SSRI treatment, has raised considerable attention and remained an unmet problem [4]. A large study using the UK General Practice Research Database of the years 1993–1999 revealed that the risk of suicidal behaviour was increased in the first 4 weeks of antidepressants, particularly during the first 1 to 9 days [52]. A similar time-frame was found by a Swedish register-based nationwide case-crossover study, reporting a peak in suicide risk during the second and third week after initiation of SSRI therapy [53]. It is therefore of particular interest that in contrast in our study, amantadine significantly reduced suicidal behaviour as early as by 2 weeks of treatment (*p* = 0.039, ES = 0.662), earlier than the total HAMD score, thereby getting beyond the above safety gap of SSRIs.

Another unmet need in the orthodox treatment of depression concerns cognitive dysfunction which impedes functional recovery in a significant proportion of MD patients [54]. Notably, in this study, we could document a significant and lasting improvement of the HAMD cluster referring to retardation (DRETARD) by amantadine as early as by 2 weeks of treatment.

Particularly the lag period of antidepressants of several weeks, increasing suicide risks before mood improvements appear, has shifted current research towards rapid-acting substances targeting the glutamatergic system. Ketamine, a non-competitive high-affinity NMDA antagonist of glutamate and licensed anaesthetic agent, has emerged as a promising candidate, since a single sub-anaesthetic intravenous dose (0.5 mg/kg) has elicited rapid improvement in depressed patients after 24 h, lasting 1–2 weeks. However, a broader application needs to address ketamine’s critical obstacles that is whether the rapid anti-depressive effects can be maintained over longer periods and self-administered application modes can be developed [58]. In view of our findings, amantadine appeared the by far more promising option compared to ketamine. Provided the early drop of suicidal behaviour holds true in larger RCTs, it could help saving patients’ lives. At least, earlier suggested adverse safety effects considering short-term psychotropic effects [5] could be ruled out. Likewise, the unexpectedly long 12-months post-study sustainability of beneficial amantadine therapy argued against mainly short-term psychotropic modes of action, the more since reduced severity and duration of further depressive episodes correlated with dropped cumulative infection measures over time (*p* < 0.001).

Notably, sustainability of amantadine therapy could also be suggested from an unexpected result of the ethically requested cross-over design of the RCT. The majority of patients who responded to amantadine treatment in Period I (Group A), maintained their response under placebo in Period II, after 14 weeks (69.2%). With respect to whole Group A, even 80% were responders after cross-over. This was doubling the primary response of patients to placebo treatment in Period I (35.3%) (Group P). Therefore, sustained amantadine efficacy may be a much more likely explanation than possible carry-over effects given the half-life of amantadine (10-31 h).

The overall clinical assessment revealed 74.2% of all study patients who benefitted from treatment after cross-over, by week 14, which exceeded response rates of 12-weeks open trials [7, 8]. Even 82.1% of patients benefitted from optional post-study amantadine treatment after 12 further months.

### BDV infection and depression

The “mood virus hypothesis” of depression [15–17] was previously supported but as yet not confirmed by a couple of findings. These were BDV-1’s ability of life-long persistence [14] in brain and blood cells of humans and many animal companions, high affinity to the hippocampus [14], behavioural and cognitive changes (animals) [14], human virus isolates [20, 21, 24], and higher prevalence data (antibodies, RNA) in psychiatric patients than controls [26–38, 40, 48]. An independent intriguing line of evidence suggested a long-term co-evolution of BDV-1 with human hosts by endogenous Borna-like N protein elements (EBLNs), integrated into the germ-line of humans and their predecessors since more than 40 million years [59, 60].

The here reported findings provided strong further support to the “mood virus hypothesis” in that dropped BDV-1 infection activity paralleled dropped depression using a drug which was shown to have anti-BDV efficacy (in vivo and in vitro) in a pilot case study [6]. However, due to amantadine’s known antidepressant effects [5], the main mode of action needs to be further clarified. Notably, amantadine had also been shown to have significant anti-manic effects in BDV-infected bipolar I or bipolar II inpatients in a pilot trial. The overall symptom reduction after 18 days was 79.2% (*p* < 0.001), and the treatment was well-tolerated [61]. Anti-manic efficacy of amantadine was unexpected given its reported pharmacological properties [5]. This argued again in favour of the drug’s anti-BDV capacity eliciting anti-manic clinical effects, even though reduced blood BDV activities could not be measured due to the too short observation period [61].

Activity measures were made available through the previous discovery of virus protein (antigen)-driven infection dynamics in any mammalian host. BDV-1 replication periods lead to excess production of antigen which induces host antibodies, both mainly forming antigen/antibody (PAG/AB) complexes circulating in blood plasma (CIC), while at the same time, levels of unbound PAG as well as AB are dropping [41].

### Infection outcomes

A modular easy-to-use EIA allowed quantitative longitudinal infection profiling of CIC, PAG, and AB throughout the trial, in parallel to clinical outcome scores. Up to almost 600 tests per assay and a mean number of 17 samples per patient were performed. In-depth analysis of the wealth of data addressed first whether and how infection variables were correlating with each other, and secondly whether and how infection and clinical variables were related. We could demonstrate a strong quantitative relationship of pre-treatment CIC and AB with PAG. Similarly validated assay results using the same EIA system were recently reported in a Lithuanian study [62]. In our trial, pre-treatment PAG- and CIC-levels correlated with AB-level reduction in the first treatment period. Notably, the initial infection load (PAG and AB) had a strong significant predictive value for short-term clinical improvement (primary response; RDHAMD) after 7 weeks. In contrast to these direct virus and host-derived variables, pre-treatment levels of their combined product (CIC) displayed no correlation to the short-term clinical improvement, but did so on the long run. Decreased CIC together with decreased PAG and AB correlated with 12-months post study clinical improvements. This remarkable finding suggested a sustainable antiviral effect against BDV-1 activity, and the likely relationship with a sustainable anti-depressive benefit.

CIC values change through antigen increase/decrease as well as antibody increase/decrease in lag time. While CIC profiles can well serve as therapy control of individual patients [15], they need to be complemented by PAG and/or AB profiles when mean values of patient groups are evaluated. Our study thus showed that patients’ blood monitoring, due to the interdependence and dynamic of BDV-1 infection variables CIC, PAG and AB, should include their complete profile, prior to, during, and after treatment. In practical terms, an important outcome of our study revealed that high PAG and AB levels in depressed patients could predict who will be likely to benefit from antiviral treatment. In so far, the study provided clear correlative evidence for a significant contribution of the infection to depression. The antiviral in vivo efficacy of amantadine had been previously demonstrated independently of clinical effects in remitted BDV-1 infected patients with affective disorders, applying a double-blind placebo-controlled design over 14 weeks. Activity-related variables (PAG and CIC) were significantly reduced (*p* = 0.028 and *p* = 0.003, respectively), as well as antibodies (AB; *p* = 0.007) [63].

CIC have been shown to be first-rate prevalence variables, superior to RNA- and antibody- only approaches. Cross-sectional studies within and between countries benefited from comparability and user friendly applicability of the BDV-CIC-EIA [64–68]. However, a general acceptance of the EIA systems is still pending even though epitope mapping and recombinant protein validated their specificity and sensitivity [47]. An attempt to question specificity reported failure to detect antigens [69], but used an inappropriate procedural approach, and did not determine through parallel experiments using recombinant proteins whether his approach met the same detection level (1.5–3.0 ng antigen/mL) achieved in our antigen-EIA [47]. The here reported study may contribute to overcome current reservations.

In conclusion, infection variables prior and post amantadine therapy were shown to be interlocked with clinical outcomes, supporting the rationale of cross-linking BDV-1 infection with depression. However, the extent to which the antidepressant efficacy of amantadine is based on its antiviral capacity against BDV-1 infection in vivo, needs to be further evaluated. This could be addressed in future studies using other antivirals with known anti-BDV-1 but lacking antidepressant effects, such as the nucleoside analogues ribavirin and favipiravir (T-705). Early ribavirin in vitro studies had used human OL cells and rat glia cells (C6) persistently infected with laboratory strains V and He/80, respectively. They found a > 90% titre decline in both cell/virus systems within 3 days, applying 20 μM ribavirin up to 13 days, but a rapid virus recovery within 2 days after drug removal [70]. Recently, favipiravir, a new potent antiviral drug against a wide spectrum of viruses, was shown to be more effective than ribavirin against BDV-1. Favipiravir rapidly reduced BDV-1 infection at 200–400 μM to almost undetectable levels by 21 days using a recombinant virus system in Vero cells, and natural virus in OL cells persistently infected with He/80 at 400 μM by 28 days [71]. Equivalent in vivo plasma levels of favipiravir (446 μM; 70 μg/mL) have been successfully applied in a lethal non-human primate model against Ebola virus disease, reducing the median viral load at day 7 by 2–3 logs [72]. In contrast to amantadine, however, the clinical application of both these drugs is associated with the risk of significant adverse effects. With respect to ribavirin, namely headache, fever, muscle pain and irritable mood were known, the latter most unfavourable in the treatment of depressed patients. Favipiravir has a risk of dose-related haemolytic anaemia, teratogenicity, and embryo-toxicity.

### In vitro findings

To further address the rationale of a mainly antiviral mode of action for amantadine, we extended in vitro efficacy studies of amantadine (1-aminoadamantane) to the closely related derivative memantine (1-amino-3,5-dimethyladamantane). Alike amantadine, memantine came into focus as low affinity non-competitive NMDA receptor antagonist with neuroprotective activity, but failed to show antidepressant effects in double-blind placebo- controlled RCTs [73, 74]. Here, we could show in vitro that amantadine was able to prevent infection (ID_50_) of rabbit cells with human BDV strain Hu-H1 [20] at concentrations as low as 2.4 to 10 ng/mL (0.005 to 0.025 μM), while memantine failed up to a 100,000 fold higher concentration (200 μg/mL; 500 μM). Interestingly, the in vitro medium dose of amantadine which abrogated replication of the same human strain by 4 weeks (0.4 μg/mL; 1 μM), corresponded exactly to the in vivo blood level achieved through the dose of 200 mg amantadine orally daily applied in patients of our trial. In contrast, laboratory-adapted str. V [14] was insensitive to amantadine treatment up to the highest dose of 1.2 μg/mL (3 μM), consistent with largely different biological properties of natural and non-natural strains [22, 23]. It should be noted that the remarkable insensitivity of BDV-1 laboratory strains observed earlier by different researchers [75–77], had quite a while led to a general questioning of amantadine’s anti-BDV efficacy. Here, we could finally dispel anybody’s doubts by demonstrating the fundamental differences between a natural human isolate and a highly adapted laboratory strain in parallel in vitro experiments. Virus-host differences had also been suggested to impact the antiviral potency of ribavirin, even though only two laboratory strains with comparable adaptations had been compared [70].

Amantadine and memantine are closely related adamantanes with not fully clarified diverse neuro-pharmacological properties [5]. Memantine neither acted as antiviral in vitro, nor elicited antidepressant efficacy above placebo [73, 74], whereas amantadine did so in this and previous open trials [6–8]. It therefore appears plausible that amantadine’s contrasting bipartite capacity may be part of a common chief mode of action, unrelated to that of memantine. Notably, experimental animal evidence suggested a key impact of BDV-1 infection on the glutamate system, targeting non-NMDA receptors of the kainate-type (KARs), specifically KA1 [78]. KARs were thought to be involved in neuronal plasticity, indispensable for memory functioning and learning [79, 80]. This would be consistent with cognitive deficiencies found earlier in BDV-1 infected rats [81]. Against this background, it appeared not too speculative that depression, amantadine, and BDV-1 infection might be linked through crosstalk at different glutamate receptor sites.

Our overall findings provided convincing evidence that oral amantadine treatment at a well-tolerated dose of 200 mg/day for at least 6 weeks was highly beneficial in MD and BD patients with BDV-1 infection, by far outweighing effect sizes of antidepressants. Amantadine impacted profoundly to safety by rapidly reducing the suicide risk earlier than SSRIs [4, 52, 53], after 2 weeks. The small study size was, however, a clear disadvantage. The cross-over design was a legitimate ethical request but implied a further disadvantage in that possible carry- over effects from the first treatment period were suggested to limit sound in-depth statistical analysis of Period II. However, unexpectedly, cross-over from amantadine to placebo resulted in maintained response in a majority of patients, arguing for favourable sustainability of amantadine therapy.

Provided large-scale studies could validate generalizability for this report, it could pave the way to a practice-changing, low cost and, in contrast to ketamine [58], easy applicable and safe mental health care perspective. Our evidence-based antiviral approach may thus also meet the needs in countries with limited resources [1].

## Conclusions

We applied the design of a double-blind placebo-controlled phase II RCT which cross-linked depression and BDV-1 infection to evaluate the antidepressant and antiviral effect of amantadine. Clinical and infection outcomes could provide the following evidence:

1. Amantadine had significant antidepressant efficacy (multi-item scores) over placebo in depressed patients with BDV-1 infection.
2. The clinical effect size (Cohen’s d-value) of amantadine tripled the overall effect size which had been calculated for orthodox antidepressants including SSRIs by independent meta-analysis.
3. Two major unsolved problems associated with orthodox treatment of depression, namely the risk of suicidal behaviour and cognitive dysfunction, were significantly decreased by amantadine as early as after the first 2 weeks.
4. Amantadine was safe at a daily dose of 200 mg and adverse events are non-significant compared to placebo.
5. Amantadine had antiviral efficacy both in infected patients and in vitro.
6. Amantadine’s antidepressant efficacy in vivo correlated with antiviral capacity against BDV-1 infection in that at least a combined antiviral/antidepressant effect was suggestive.

These findings supported the paradigm of a contributory role of BDV-1 infection in depression. Screening for BDV-1 infection using easy accessible serum or plasma samples could open the window to a new very cost effective treatment option suitable for depressed infected patients. Our study clearly showed the benefit of amantadine for these patients exceeding that of orthodox antidepressants with respect to effect size and safety. Our findings thus anticipate a novel possibly practice-changing mental health care perspective for depressed BDV-1 infected patients which may even be applicable at a global scale. At least, our novel approach could inspire the conduct of large-scale clinical studies which are needed to further examine the generalizability of our findings.

## Supplementary information

**Additional file 1.** Trial registration.

**Additional file 2.** Study history and disclaimer.

**Additional file 3: Table S1.** Overall clinical response.

**Additional file 4: Figure S1.** Cumulative effect size development.

**Additional file 5: Table S2.** Reported single adverse effects.

**Additional file 6:** Total treatment outcome (RDHAMD week 7) correlated with pre-treatment antibody load (**Figure S2**) and antigen load (**Figure S3**).

**Additional file 7: Figure S4.** In vitro inhibition of replication comparing human and laboratory strains.

## Data Availability

The datasets generated and/or analysed during the current study are not publicly available due to protection of individual privacy of participants, but are available from the corresponding authors on reasonable request.

## Abbreviations

AB: Antibodies
BD: Bipolar disorder
BDV: Borna disease virus
BfS: Mental health self-rating score (von Zerssen)
CIC: BDV circulation immune complexes
DALYs: Disability-adjusted life years
EIA: Enzyme immune assay
ES: Effect size (Cohen’s d)
FDA: Food and Drug Administration
HAMD: Hamilton rating scale for depression
IFT: Indirect immune fluorescence test
KARs: Kainate receptors
mABs: Monoclonal antibodies
MD: Major depression
MHH: Hannover Medical School
NMDA: N-methyl-D-aspartate
OL: Human oligodendroglia
PAG: BDV antigens (N, P proteins, N/P heterodimer)
PBMCs: Peripheral blood mononuclear cells
PBS: Phosphate-buffered saline
RCT: Randomized clinical trial
RNA: Ribonucleic acid
SCL-90R: Self-rating symptoms checklist
SSRIs: Serotonin reuptake inhibitors
WHO: World Health Organization
YRS: Young rabbit spleen
