# Supplementary material for: Antiviral treatment perspective against Borna disease virus 1 infection in major depression: a double-blind placebo-controlled randomized clinical trial
Source: BMC Pharmacol Toxicol. 2020 Feb 17;21:12. doi: 10.1186/s40360-020-0391-x (PMC7027224; doi:10.1186/s40360-020-0391-x)
Supplement: Supplementary file 1 — Additional file 1. Trial registration. [file 40360_2020_391_MOESM1_ESM.pdf]

PLEASE NOTE: *This trial has been registered retrospectively.*

Trial Description

Title

An investigation of the clinical efficacy of amantadine on depressive syptoms and cognitive deficits in Borna Disease Virus (BDV) infected patients with an affective psychosis; a prospective, randomized, double-blind, placebo controlled, monocentric study.

Trial Acronym

[---]*

URL of the trial

[---]*

Brief Summary in Lay Language

The study examines the efficacy of amantadine on depressive symptoms and cognitive (i.e. concerning thinking and perception) deficits in depressed patients who are infected with Borna disease virus (BDV). Potentially pre-existing drug treatments are continued and the study participants receive in a first phase amantadine or placebo. In a second phase, those previously treated with amantadine receive a placebo and vice versa. The course of the mentioned symptoms is repeatedly investigated.

Brief Summary in Scientific Language

Amantadine sulfate will be evaluated as add-on treatment for the antidepressant and phase prophylactic treatment of acute depressive episodes in bipolar or recurrent depressive affect disorders for a potential reduction of depressive symptoms and BDV activity. It is a mono-centric, prospective, randomized, double-blind, placebo-controlled cross-over study. In the Study, it was planned to include 20-40 inpatiets (at inclusion) and 20-40 outpatients.

Organizational Data

- DRKS-ID: **DRKS00007649**
- Date of Registration in DRKS: **2015/03/04**
- Date of Registration in Partner Registry or other Primary Registry: **[---]***
- Investigator Sponsored/Initiated Trial (IST/IIT): **yes**
- Ethics Approval/Approval of the Ethics Committee: **Approved**
- (leading) Ethics Committee Nr.: **1508-1997 , Ethikkommission der Medizinischen Hochschule Hannover**

Secondary IDs

Health condition or Problem studied

- ICD10: **F31.3 - Bipolar affective disorder, current episode mild or moderate depression**
- ICD10: **F31.4 - Bipolar affective disorder, current episode severe depression without psychotic symptoms**
- ICD10: **F32.1 - Moderate depressive episode**
- ICD10: **F33.1 - Recurrent depressive disorder, current episode moderate**
- ICD10: **F33.2 - Recurrent depressive disorder, current episode severe without psychotic symptoms**

Interventions/Observational Groups

- Arm 1: **200 mg of amantadine or placebo as add-on treatment. Cross-over after six weeks.**
- Arm 2: **200 mg of amantadine or placebo as add-on treatment. Cross-over after six weeks.**

Characteristics

- Study Type: **Interventional**
- Study Type Non-Interventional: **[---]***
- Allocation: **Randomized controlled trial**
- Blinding: **[---]***
- Who is blinded: **patient/subject, investigator/therapist, data analyst**
- Control: **Placebo**
- Purpose: **Treatment**
- Assignment: **Crossover**
- Phase: **II**
- Off-label use (Zulassungsüberschreitende Anwendung eines Arzneimittels): **N/A**

Primary Outcome

Change of HAMD rating results after 6 weeks of treatment and BDV activity comparing amantadin and placebo group

Secondary Outcome

Beck Depression Inventory (BDI)
von Zerssen's Mental State Scale (Bf-S)
Symptom Checklist (SCL)
Global Assessment Scale (GAS)
Cognitive Functions

Countries of recruitment

- DE **Germany**

Locations of Recruitment

- University Medical Center **Medizinische Hochschule Hannover, Hannover**

Recruitment

- Planned/Actual: **Actual**
- (Anticipated or Actual) Date of First Enrollment: **1997/04/28**
- Target Sample Size: **40**
- Monocenter/Multicenter trial: **Monocenter trial**
- National/International: **National**

Inclusion Criteria

- Gender: **Both, male and female**
- Minimum Age: **30 Years**
- Maximum Age: **70 Years**

Additional Inclusion Criteria

Acutely depressed patients requiring treatment, according to the ICD-10 classification: F31.3, 31.4, 31.5 as well as F33.0, F33.1, F33.2, F33.3 and the respective DSM-IV criteria

Men and women between 30 and 70 years of age

Informed consent of the patient

Exclusion criteria

Suspected exogenous depression

Hypersensitivity against ingredients of amantadine or the placebo

Conditions in which the use of amantadine is contraindicated (e.g. clinically relevant renal insufficiency)

Other severe systemic conditions (e.g. cardiovascular conditions, blood coagulation disorders, malignancies requiring palliative care, collagenoses or florid infections)

Current pregnancy or breastfeeding

Compulsory in-patient treatment

Unability to give informed consent

Addresses

- **Primary Sponsor**

  Medizinische Hochschule Hannover
  Carl-Neuberg-Str. 1
  30625 Hannover
  Germany

  Telephone: **[---]***
  Fax: **[---]***
  E-mail: **[---]***
  URL: **www.mh-hannover.de**

- **Contact for Scientific Queries**

  Medizinische Hochschule Hannover
  *** ***
  Carl-Neuberg-Str. 1
  30625 Hannover
  Germany

  Telephone: *****
  Fax: **[---]***
  E-mail:
  4968435962307a4f324c38316f777573484b79654f43694e654453484c3237333 77534367164342b5970633d at drks.de
  URL: **www.mh-hannover.de**

- **Contact for Public Queries**

  Medizinische Hochschule Hannover
  *** ***
  Carl-Neuberg-Str. 1
  30625 Hannover
  Germany

  Telephone: *****

Contact for Public Queries

Medizinische Hochschule Hannover
*** ***
Carl-Neuberg-Str. 1
30625 Hannover
Germany

Telephone: *****
Fax: **[---]***
E-mail:
4968435962307a4f324c38316f777573484b79654f43694e654453484c3237333 77534367164342b5970633d at drks.de
URL: **www.mh-hannover.de**

Sources of Monetary or Material Support

- **Institutional budget, no external funding (budget of sponsor/PI)**

Medizinische Hochschule Hannover
Carl-Neuberg-Strasse 1
30625 Hannover
Germany

Telephone: **[---]***
Fax: **[---]***
E-mail: **[---]***
URL: **[---]***

Status

- Recruitment Status: **Recruiting complete, follow-up complete**
- Study Closing (LPLV): **1999/06/18**

Trial Publications, Results and other documents

** This entry means the parameter is not applicable or has not been set.*
**** This entry means that data is not displayed due to insufficient data privacy clearing.*
