# Supplementary material for: Antiviral treatment perspective against Borna disease virus 1 infection in major depression: a double-blind placebo-controlled randomized clinical trial
Source: BMC Pharmacol Toxicol. 2020 Feb 17;21:12. doi: 10.1186/s40360-020-0391-x (PMC7027224; doi:10.1186/s40360-020-0391-x)
Supplement: Supplementary file 2 — Additional file 2. Study history and disclaimer. [file 40360_2020_391_MOESM2_ESM.pdf]

## Additional file 2: Study history and disclaimer

Two decades of pre-publication history are as unusual as the study's topic is unique. No similar study has been conducted in the meantime, while our study's novelty and timeliness remained and even increased up to now.

### Time-frame of the study

The core part of the clinical trial including preparatory parts and basic virology was conducted between 1997 and 1999. A pilot virological screening phase started on 27th of January 1997. The date of first enrolment was on 28th of April 1997. Post-phase virological testing periods as well as clinical and virological evaluation updates were extended up to 2002. Final statistical analysis updates were done until 2010. The trial was retrospectively registered on 4th of March 2015 (see Additional file 1). The discontinuity in post-study evaluation of clinical and virological study parts, study statistics and manuscript versions up to the current final form was on the one hand due to shifting of workplace and/or area of activity of study authors. On the other hand, completion and publication was significantly impeded through doubts raised in the scientific community about human infection until final incontrovertible proof of human infection by BDV-1 caused encephalitis cases published in 2018.

### Collaborative work and institutions

Throughout its core part and time-frame, the study was conducted as collaborative work, independent of company-sponsoring. Psychiatrists (DED, HME, CWS) of the Department of

Clinical Psychiatry and Psychotherapy (presently Department of Mental Health) together with the Chief Medical Statistician (HH) of the Department of Biometrics at the Hannover Medical School (MHH), Hanover, Germany, collaborated with virologists of the Institute of Virology at the Free University of Berlin (FU Berlin) (HL) and of the Project Bornavirus Infections (LB) at the Robert Koch Institute (RKI), Berlin, Germany. DED has later moved to a private psychiatric hospital, the Burghof-Clinic, Rinteln, Germany (present address). CWS has moved to a pharmaceutical company. His current operating level is unrelated to any aspect of this trial. HL, HME, and HH have meanwhile the status of a professor emeritus at their home universities. HME passed away on 16th of September 2018, but approved a complete pre-final version of the manuscript. LB has recently left the RKI upon retirement.

**Disclaimer (LB)**

The main article and additional files providing supporting information reflect the author's opinion but not the opinion of the Robert Koch Institute (RKI). All virological work assigned to the study has been performed during run time of the project Bornavirus infections at RKI laboratories. The project was terminated upon RKI decision by 31st December 2005. LB shifted to another department at RKI and worked projects completely unrelated to and separated from her former BDV research. Therefore, since 2006, any publications reflecting this former work included the above disclaimer and/or were affiliated to institutions independent of her tenure at RKI. Upon retirement she has reassigned to an unaffiliated authorship. For the sake of formality in collaborative studies, she assigned to the Freelance Bornavirus Workgroup of Joint Senior Scientists which HL has founded after becoming professor emeritus.
