# Supplementary material for: Antiviral treatment perspective against Borna disease virus 1 infection in major depression: a double-blind placebo-controlled randomized clinical trial
Source: BMC Pharmacol Toxicol. 2020 Feb 17;21:12. doi: 10.1186/s40360-020-0391-x (PMC7027224; doi:10.1186/s40360-020-0391-x)
Supplement: Supplementary file 3 — Additional file 3: Table S1. Overall clinical response. [file 40360_2020_391_MOESM3_ESM.pdf]

Additional file 3:

Table S1. Overall clinical response.

**Group A**	Period I (week 7)	Period II (week 14)	Follow-up (week 66)
Patient code	Amantadine	Placebo	Amantadine optional
01-01-001	NR	R	NR
02-04-001	NR	R	R
03-06-001	R	R	R
04-12-001	NR	R	NR
05-01-002	R	R	R
06-03-002	R	R	R
07-18-002	R	**drop out**	**drop out**
08-19-002	R	NR	NR
09-07-001	R	R	R
10-10-001	R	R	R
11-13-001	R	R	R
12-16-001	R	NR	R
13-06-002	R	R	R
14-10-002	R	R	**drop-out**
15-12-002	R	R	R
16-15-002	R	NR	NR
Total R	13	12	10
Total NR	3	3	4
**Group P**	Period I (week 7)	Period II (week 14)	Follow-up (week 66)
Patient code	Placebo	Amantadine	Amantadine optional
01-05-001	R	R	R
02-08-001	NR	**drop-out**	**drop-out**
03-09-001	NR	NR	R
04-02-002	NR	NR	R
05-08-002	NR	R	R
06-20-002	NR	NR	R
07-22-002	NR	R	**drop-out**
08-23-002	R	R	R
09-02-001	NR	R	R
10-03-001	R	R	NR
11-11-001	NR	R	**drop-out**
12-14-001	R	R	R
13-04-002	NR	NR	R
14-09-002	NR	R	R
15-11-002	NR	R	R
16-16-002	R	R	R
17-17-002	R	NR	R
Total R	6	11	13
Total NR	11	5	1

Study patients were randomly assigned to either Group A starting with amantadine or Group P starting with placebo. Last digits of patient codes indicate out-patients (1) and in-patients (2). R = clinical responder, NR= clinical non-responder. Clinical response was defined as reduction of the pre-treatment HAMD score of ≥ 25% by week 7 (main outcome), after cross-over by week 14, and after further 12 months follow-up optional amantadine treatment by week 66.
