# Supplementary material for: Antiviral treatment perspective against Borna disease virus 1 infection in major depression: a double-blind placebo-controlled randomized clinical trial
Source: BMC Pharmacol Toxicol. 2020 Feb 17;21:12. doi: 10.1186/s40360-020-0391-x (PMC7027224; doi:10.1186/s40360-020-0391-x)
Supplement: Supplementary file 4 — Additional file 4: Figure S1. Cumulative effect size development. [file 40360_2020_391_MOESM4_ESM.pdf]

Additional file 4:

Figure S1. Cumulative effect size development of amantadine vs. placebo Groups (Cohen's d-values).

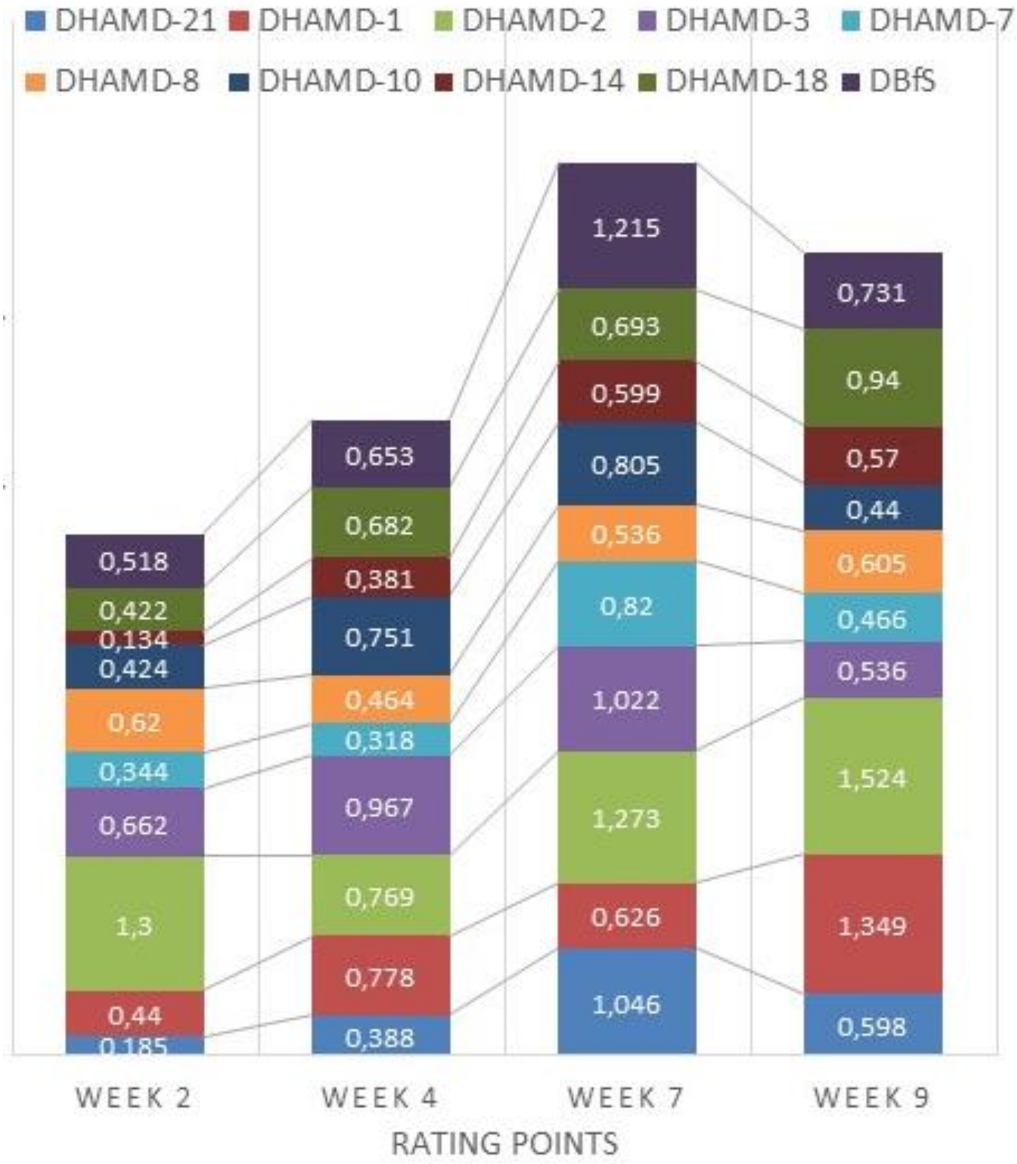

Cohen's d-values of clinical significance: values of 0.2, 0.5, and 0.8 were proposed to represent small, medium, and large effects, respectively.

D=Difference scores of
HAMD-21 Hamilton rating scale of depression; 21 items. Single items:
HAMD-1 "depressed mood"; **HAMD-2** "feeling of guilt; **HAMD-3** "suicide"; **HAMD-7** "work and activities"; **HAMD-8** "retardation"; **HAMD-10** "anxiety (psychic)"; **HAMD-14** "sexual function"; **HAMD-18** "day-variations";
BfS "Befindlichkeitsskala nach von Zerssen (self-rating well-being scale)"
