# Supplementary material for: Antiviral treatment perspective against Borna disease virus 1 infection in major depression: a double-blind placebo-controlled randomized clinical trial
Source: BMC Pharmacol Toxicol. 2020 Feb 17;21:12. doi: 10.1186/s40360-020-0391-x (PMC7027224; doi:10.1186/s40360-020-0391-x)
Supplement: Supplementary file 5 — Additional file 5: Table S2. Reported single adverse effects. [file 40360_2020_391_MOESM5_ESM.pdf]

**Additional file 5:**

## Table S2. Reported single adverse effects.

| | Amantadine (N = 33*) | Placebo (N = 33*) |
|---|---|---|
| No. of patients with adverse events | 15 | 11 |
| No. of patients without adverse events | 18 | 22 |
| **Total No. of Events Reported** | **29** | **23** |
| Dry of mouth | 4 | 4 |
| Restlessness | 3 | 1 |
| Abdominal, pain | 3 | |
| Headache | 1 | 2 |
| Constipation | 2 | 2 |
| Diarrhoea / abdominal irritation | 2 | 2 |
| Nausea | | 3 |
| Dizziness | 2 | |
| Bodily exhaustion | 1 | 1 |
| Difficulty Urinating | 1 | |
| Skin rash | 1 | |
| Tremor | 1 | 2 |
| Sweating | 2 | 1 |
| Sleep disturbances | 1 | |
| Pollacisuria | 1 | 2 |
| Tachycardia/Palpitation | 1 | 1 |
| Back pain | 1 | 2 |
| Cold Hands | 1 | |
| Weakness of concentration | 1 | |

* Two of the 33 patients did not participate in/finish the second treatment period. Drop-outs were unrelated to adverse side-effects.
