# Supplementary material for: Antiviral treatment perspective against Borna disease virus 1 infection in major depression: a double-blind placebo-controlled randomized clinical trial
Source: BMC Pharmacol Toxicol. 2020 Feb 17;21:12. doi: 10.1186/s40360-020-0391-x (PMC7027224; doi:10.1186/s40360-020-0391-x)
Supplement: Supplementary file 6 — Additional file 6: Total treatment outcome (RDHAMD week 7) correlated with pre-treatment antibody load (Figure S2) and antigen load (Figure S3). [file 40360_2020_391_MOESM6_ESM.pdf]

Additional file 6:

Figure S2 Correlation of total treatment outcome (RDHAMD week 7) with pre-treatment BDV-1 antibody load.

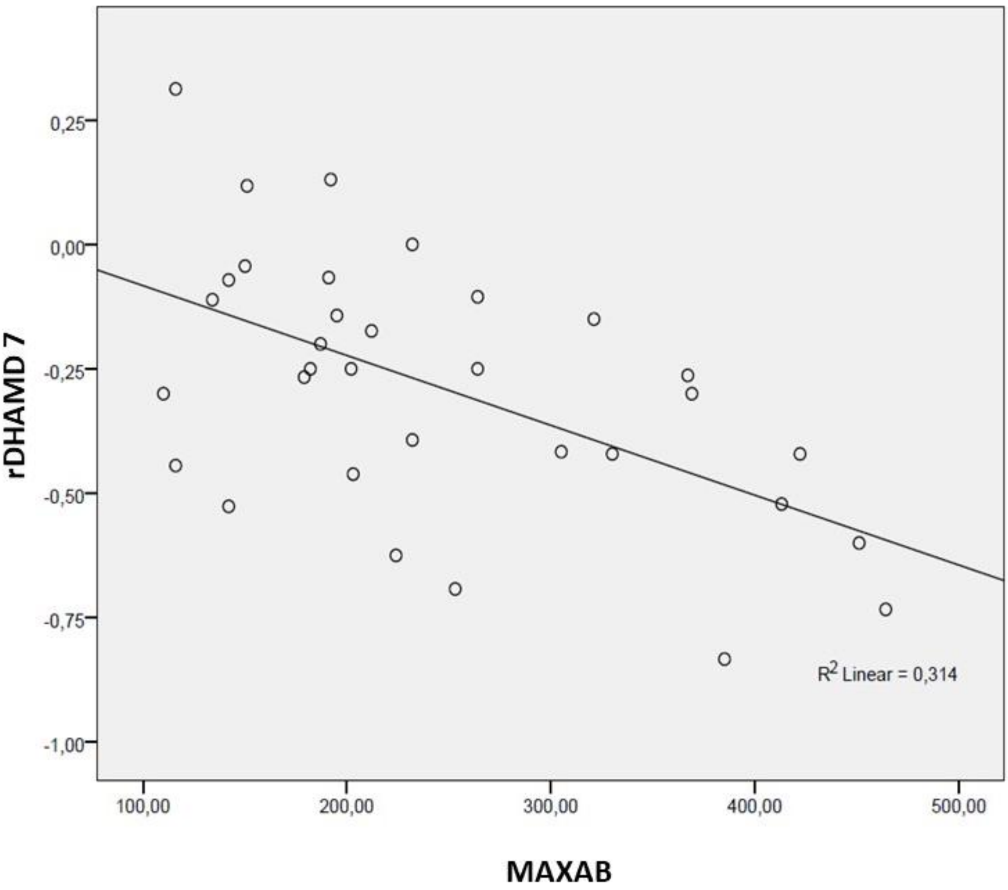

Total treatment outcome (amantadine and placebo)
rDHAMD7 = relative differences of the HAMD scores on week 7
MAXAB = pre-treatment maximum values of BDV-1 antibodies in plasma

Figure S3 Correlation of total treatment outcome (RDHAMD week 7) with pre-treatment BDV-1 antigen load.

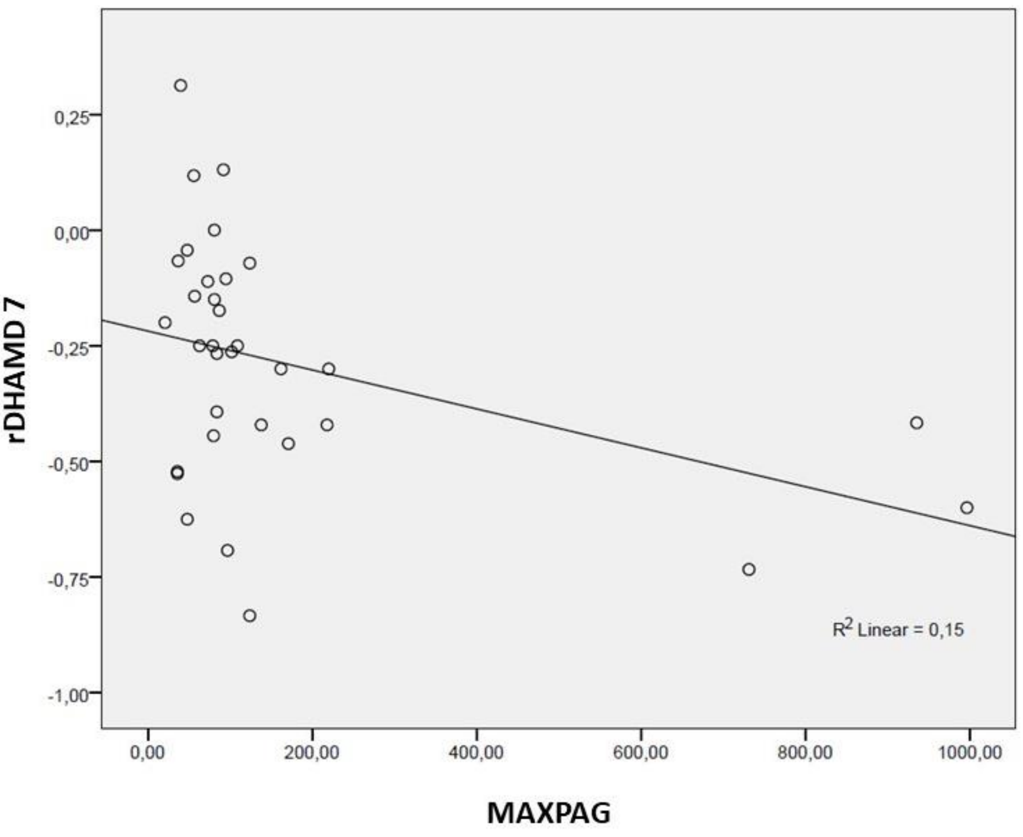

Total treatment outcome (amantadine and placebo)
rDHAMD7 = relative differences of the HAMD scores on week 7
MAXPAG = pre-treatment maximum values of BDV-1 antigens in plasma
