# Supplementary material for: Antiviral treatment perspective against Borna disease virus 1 infection in major depression: a double-blind placebo-controlled randomized clinical trial
Source: BMC Pharmacol Toxicol. 2020 Feb 17;21:12. doi: 10.1186/s40360-020-0391-x (PMC7027224; doi:10.1186/s40360-020-0391-x)
Supplement: Supplementary file 7 — Additional file 7: Figure S4. In vitro inhibition of replication comparing human and laboratory strains. [file 40360_2020_391_MOESM7_ESM.pdf]

Additional file 7:

Figure S4. ***In vitro* inhibition of replication comparing human and laboratory strains of BDV-1**

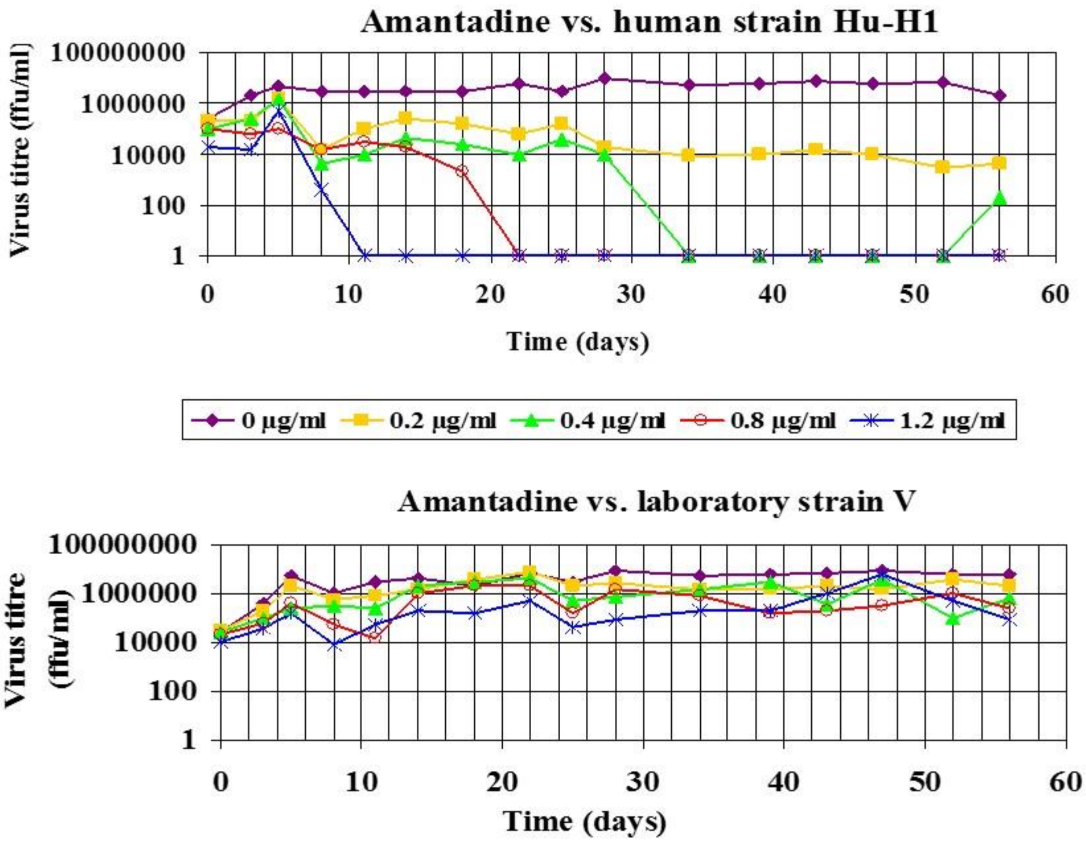

Inhibition-of-replication longitudinal assay (60 days) *in vitro*, testing the antiviral efficacy of different amounts of amantadine (0 µg mL^{-1} to 1.2 µg mL^{-1}) against BDV-1 human strain Hu-H1 compared to laboratory adapted str. V of animal origin, using the same cell type (human oligodendroglial [OL] cells).
